# Structural mechanism of synergistic activation of Aurora kinase B/C by phosphorylated INCENP

**DOI:** 10.1038/s41467-019-11085-0

**Published:** 2019-07-18

**Authors:** Kamal R. Abdul Azeez, Sneha Chatterjee, Channing Yu, Todd R. Golub, Frank Sobott, Jonathan M. Elkins

**Affiliations:** 1 Structural Genomics Consortium, University of Oxford, Old Road Campus Research Building, Old Road Campus, Roosevelt Drive, Oxford, OX3 7DQ UK; 2 Biomolecular and Analytical Mass Spectrometry, University of Antwerp, Groenenborgerlaan 171, 2020 Antwerp, Belgium; 3 Broad Institute, 415 Main St, Cambridge, MA 02142 USA; 4 Dana-Farber Cancer Institute, 450 Brookline Ave, Boston, MA 02215 USA; 5 Astbury Centre for Structural Molecular Biology, University of Leeds, Woodhouse Lane, Leeds, LS2 9JT UK; 6 School of Molecular and Cellular Biology, University of Leeds, Woodhouse Lane, Leeds, LS2 9JT UK; 7 Structural Genomics Consortium, Universidade Estadual de Campinas, Cidade Universitária Zeferino Vaz, Av. Dr. André Tosello, 550, Barão Geraldo, Campinas, SP 13083-886 Brazil

**Keywords:** Kinases, Kinases, X-ray crystallography

## Abstract

Aurora kinases B and C (AURKB/AURKC) are activated by binding to the C-terminal domain of INCENP. Full activation requires phosphorylation of two serine residues of INCENP that are conserved through evolution, although the mechanism of this activation has not been explained. Here we present crystal structures of the fully active complex of AURKC bound to INCENP, consisting of phosphorylated, activated, AURKC and INCENP phosphorylated on its TSS motif, revealing the structural and biochemical mechanism of synergistic activation of AURKC:INCENP. The structures show that TSS motif phosphorylation stabilises the kinase activation loop of AURKC. The TSS motif phosphorylations alter the substrate-binding surface consistent with a mechanism of altered kinase substrate selectivity and stabilisation of the protein complex against unfolding. We also analyse the binding of the most specific available AURKB inhibitor, BRD-7880, and demonstrate that the well-known Aurora kinase inhibitor VX-680 disrupts binding of the phosphorylated INCENP TSS motif.

## Abstract (web summary)

The inner centromere protein (INCENP) activates Aurora kinase B (AURKB) and Aurora kinase C. Here the authors provide insights into the activation mechanism of AURKB/C by determining the crystal structure of fully active phosphorylated human AURKC bound to the phosphorylated C-terminal IN-box section of human INCENP.

## Introduction

There are three Aurora kinases in mammals, Aurora kinase A, Aurora kinase B and Aurora kinase C (AURKA, AURKB and AURKC). Although the kinase domains of the Aurora kinases have significant homology (AURKC has 84% and 68% sequence identity to AURKB and AURKA, respectively) and their ATP binding sites are 100% conserved, they have different regulatory mechanisms and perform different functions. AURKA and AURKB catalyse critical phosphorylation events in mitosis while AURKC is mainly expressed in gametes and is important for meiosis: micro-injection of inactive AURKC into mouse oocytes (in which AURKB protein was not detected) caused cytokinesis failure in meiosis I, with errors in chromosome alignment and segregation^1^. In a similar manner, an AURKC ATP binding site mutant injected into mouse oocytes also caused arrest at meiosis I for the majority of oocytes, although some did proceed through cytokinesis normally^2^. This suggested that, as well as the regulation of the spindle assembly checkpoint in meiosis by an AURKC chromosomal passenger complex (CPC), there is also the possibility for an AURKB CPC to perform this function^2^. However, the oocytes that did proceed despite lack of AURKC activity were aneuploid, showing the importance of AURKC for chromosome segregation in meiosis. It has been suggested that AURKC compensates for lack of AURKB during maturation of the early embryo and that it achieves this through greater protein stability during the single cell stage (meiosis) and in early cell divisions^3^. Indeed, AURKB but not AURKC was shown to be dispensable during early cell divisions^4^. AURKC lacks some protein degradation markers found in AURKB and so one current model is that AURKC provides a longer lasting version of AURKB that will be present through meiosis. In artificial systems AURKC is able to substitute for AURKB in mitosis^5,6^, suggesting again that it is the regulation of AURKB and AURKC protein levels which is important rather than intrinsic differences between the kinase domains.

AURKB is found in the CPC together with the inner centromere protein (INCENP), borealin and survivin^7–9^. Formation of the CPC and correct localisation of the CPC is essential for a properly ordered sequence of events in the exit from mitosis. AURKC is also found in the CPC and is also activated by INCENP^10^. AURKB and AURKC are bound to the C-terminal “IN-box” region of INCENP^11^. This IN-box region contains a conserved C-terminal TSS motif; in *C. elegans* this motif (GSS in *C. elegans)* is a substrate of AURKB (AIR-2) and TSS motif phosphorylation increases AURKB activity^12^. Similar results were obtained with human AURKB and INCENP^13^. These two phosphorylation sites are conserved through evolution^11,12^ pointing to an essential and fundamental role of the TSS motif. Previous crystal structures of human or *X. laevis* AURKB bound to INCENP revealed an extensive interaction of AURKB with the C-terminal IN-box region^14,15^. However, these previous structures were of partially active AURKB:INCENP complexes lacking a phosphorylated TSS motif and thus the mechanistic role of this evolutionarily conserved motif has not been explained.

To understand better the molecular mechanisms of regulation of AURKB and AURKC by INCENP we determined structures of fully active (phosphorylated) human AURKC bound to the phosphorylated C-terminal IN-box section of human INCENP (residues 835–903). These structures demonstrate the structural changes upon activation and the roles of essential conserved residues. We show that the TSS motif phosphorylations activate AURKB and AURKC by changing the *K*_M_ for substrate. In addition, we present crystallographic and biophysical characterisation of a potent and extremely selective AURKB and AURKC inhibitor^16^.

## Results

### Structure determination of activated human AURKC:INCENP

**Fig. 1:**
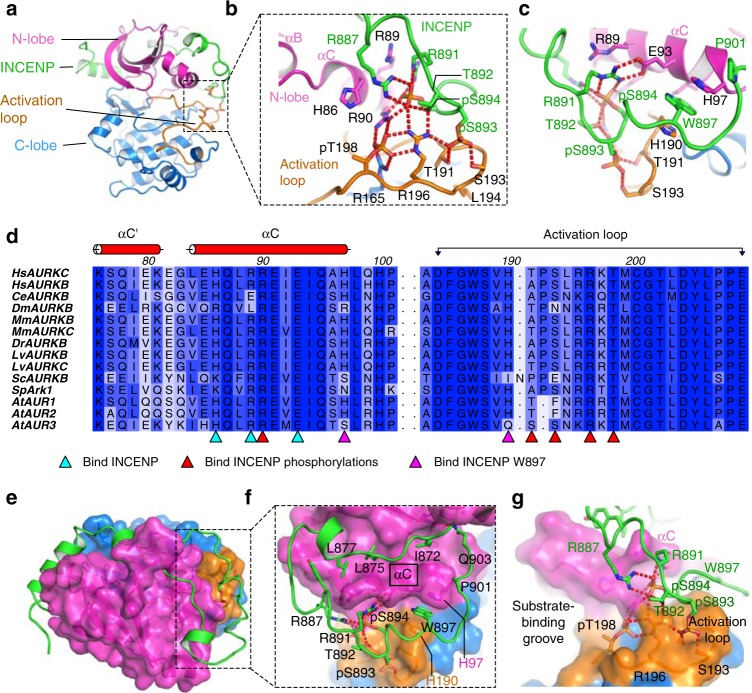
Phosphorylated INCENP binds the activation loop of Aurora kinase B/C. **a** Structure of activated AURKC bound to the phosphorylated INCENP “IN-box”. **b**, **c** Views from two angles showing how the phosphorylated INCENP makes extensive hydrogen-bonding interactions with the activation loop and αC-helix of AURKC, and contributes significantly to arranging the active conformation and substrate binding region of the kinase. **d** Sequence alignment of the αC and activation loop regions of AURKB or AURKC from diverse organisms. Residues involved in binding INCENP are marked with triangles below the alignment. For a full sequence alignment and sequence accession numbers see Supplementary Fig. 4. The sequence alignment was created using ClustalO^46^ and Aline^47^. **e** In the fully active AURKC:INCENP structure INCENP makes contact with the Aurora kinase N-lobe and C-lobe, and the activation loop. **f**, **g** Views from two angles showing how INCENP binds on two sides of AURKC αC including an aromatic stacking interaction between INCENP W897 and AURKC H97 and H190, as well as the hydrogen-bonding network around INCENP pS893 and pS894. These binding interactions link AURKC αC and activation loop into a stable conformation, and the INCENP phosphorylations help to form the substrate binding groove, thus activating AURKC to different substrates

To initiate structural and biochemical studies on AURKB and AURKC INCENP complexes we constructed plasmids for bi-cistronic co-expression of the INCENP IN-box (residues 835–903) with AURKB or AURKC kinase domains. Various truncations of AURKB and AURKC were assessed for production of soluble and stable Aurora:INCENP complexes with several of each producing high yields of soluble protein (Supplementary Table 1). In all purified protein complexes, electrospray ionisation mass spectrometry revealed two stoichiometric phosphorylations of INCENP, a sub-stoichiometric third INCENP phosphorylation, and 1–3 phosphorylations of AURKB or AURKC (Supplementary Fig. 1). Several of the phosphorylated AURKC:INCENP protein complexes were used in crystallisation trials together with the ATP-competitive dual AURKB/AURKC-specific inhibitor BRD-7880^16^. A complex containing residues 36–305 of AURKC isoform 1 gave crystals which diffracted to 1.75 Å resolution (Table 1). It was possible to solve the structure from this data by the molecular replacement method using a model of human AURKB^15^ and then to build and refine a model of AURKC:INCENP. The final model contains residues 36–303 of AURKC and residues 838–903 of INCENP (Fig. 1a). The electron density clearly showed that AURKC residues Thr39 and Thr198 were phosphorylated, as were INCENP residues Ser893 and Ser894 (Supplementary Fig. 2). The third INCENP phosphorylation site was not identified and since all INCENP serine or threonine residues were clearly resolved in the electron density it may be that crystallisation selected only the AURKC:INCENP complexes without the third INCENP phosphorylation.

**Table 1:** Data collection and refinement statistics

	AURKC: INCENP: BRD-7880	AURKC: INCENP: VX-680	AURKA: BRD-7880
PDB ID	6GR8	6GR9	6GRA
Space group	*P*2_1_2_1_2_1_	*P*3_1_21	*P*6_1_22
No. of molecules in the asymmetric unit	1	1	1
Unit cell dimensions *a*, *b*, *c* (Å), α, β, γ (°)	56.7, 72.5, 83.1 90.0, 90.0, 90.0	82.6, 82.6, 121.0 90.0, 90.0, 120.0	83.2, 83.2, 171.9 90.0, 90.0, 120.0
Data collection			
Resolution range (Å)^a^	56.65–1.75 (1.78–1.75)	71.55–2.25 (2.32–2.25)	85.93–2.60 (2.74–2.60)
Unique observations^a^	34077 (1433)	23264 (2104)	11497 (1603)
Average multiplicity^a^	5.4 (4.1)	5.2 (4.9)	8.6 (8.3)
Completeness (%)^a^	97.1 (77.3)	100.0 (100.0)	100.0 (100.0)
* R*_merge_^a^	0.10 (0.58)	0.07 (0.66)	0.07 (0.77)
Mean ((*I*)/σ(*I*))^a^	9.8 (2.1)	14.8 (2.0)	13.3 (2.4)
Mean CC(1/2)	0.995 (0.709)	0.999 (0.689)	0.999 (0.818)
Refinement			
Resolution range (Å)	54.61–1.75	71.55–2.25	66.52–2.60
* R*-value, *R*_free_	0.18, 0.22	0.18, 0.22	0.21, 0.27
r.m.s. deviation from ideal bond length (Å)	0.008	0.008	0.008
r.m.s. deviation from ideal bond angle (°)	1.35	1.33	1.29
Ramachandran outliers^b^ most favoured	0.31% 97.5%	0.00% 96.03%	0.00% 97.15%
Average *B*-factors (protein, ligand, solvent) (Å^2^)	19, 16, 28	46, 40, 42	80, 73, 63

^a^Values within parentheses refer to the highest resolution shell

^b^Values from molprobity

### The phosphorylated TSS motif binds the AURKC activation loop

AURKC Thr198 is the canonical activation loop residue that in many Ser/Thr kinases is phosphorylated as part of the activation mechanism of the kinase. Phosphorylation of kinases at this conserved site causes the activation loop to adopt a more ordered arrangement that forms part of the substrate binding surface. The phosphorylated Thr198 (pThr198) is bound as expected by the side-chain of Arg165 from the kinase’s HRD motif (Fig. 1b), a feature common to the activation mechanism of protein kinases. pThr198 is also bound by the side-chains of Arg90 from the αC helix, a feature in common with activated AURKA:TPX2^17^, and Arg196 from the activation loop.

Unexpectedly, Arg196 also forms hydrogen bonds with both of the phosphorylated INCENP residues pS893 and pS894 (Fig. 1b, c). INCENP pS893 forms hydrogen bonds with AURKC Arg196, Thr191 and Ser193 which are all located on the activation loop. INCENP pS894 forms hydrogen bonds with AURKB Arg196 and Arg90, as well as with INCENP Arg887 and Arg191. All of the AURKC activation loop residues (184–209) except Thr191 are conserved in AURKB (Fig. 1d and Supplementary Figs. 3 and 4) and so it is very likely that AURKB has a similar activation loop conformation in its active form. Of particular interest, with the exception of Thr191, all AURKC residues involved in binding INCENP pS893 and pS894 are highly conserved through evolution (Fig. 1d), even in organisms such as *Saccharomyces cerevisiae* and *Arabidopsis thaliana* where the putative INCENP protein lacks the TSS motif (Supplementary Fig. 5). Furthermore, other adjacent INCENP residues are also highly conserved, and these have conserved binding positions on AURKC. In particular, INCENP Trp897 is bound in an aromatic stacking interaction by AURKC His97 and His190; these AURKC residues are conserved except where the INCENP equivalent does not have a corresponding tryptophan. Arg891 forms salt bridges with both pS894 and Glu93 from αC of AURKC (Fig. 1) which appears to be an essential interaction in the binding of the TSS motif. Just two of the residues that appear to be important for INCENP TSS motif binding are not highly conserved: Arg887 which binds pS894 (Fig. 1) is conserved only in the higher mammals, which have an AURKC enzyme. The same is also true for the adjacent Tyr888 which binds against AURKC αC and forms a hydrogen bond with Glu85.

The AURKC conformation around pThr198 is broadly similar to that seen in active AURKA^17^ (Supplementary Fig. 6), although with a different conformation of Arg196 to allow Arg196 to form hydrogen bonds simultaneously to pThr198 and INCENP pSer893 and pSer894 (Fig. 1b), and crucially with a flip of the backbone conformation at residues 193–195 which directs Leu194 away from INCENP (Fig. 1b). In previous structures of *Xenopus laevis* AURKB:INCENP^14^, which have a phosphorylated activation loop but lack the C-terminal region of INCENP, the AURKB residue equivalent to AURKC Leu194 is directed towards INCENP, and is in a position incompatible with binding of INCENP pS893 (Supplementary Fig. 6). Thus, the data is consistent with a mechanism where binding of INCENP pS893 and pS894 promotes a flip of the activation loop at Leu194, a change in conformation of Arg196, and hydrogen-bonding of pThr198 with Arg90. The conformation of the activation loop in the *Xenopus laevis* AURKB structures can therefore be considered as partially activated. In our previous structure of partially active *Homo sapiens* AURKB:INCENP^15^, which lacks phosphorylation on the activation loop, the activation loop formed a domain-swap dimer, made more favourable by hydrophobic interactions involving AURKB Met249; such domain-swaps can be a mechanism of trans-phosphorylation during activation^18^. AURKB Met249 is not conserved in AURKC (Supplementary Fig. 3), and so it seems less likely that there could be such a domain-swap arrangement for inactive AURKC. Nevertheless it remains possible that both AURKC and AURKB are activated by *trans*-phosphorylation of their activation loops and either *cis* or *trans-*phosphorylation of INCENP.

Compared with partially active AURKB:INCENP^15^ a much longer piece of the INCENP IN-box is in an ordered conformation and bound to the kinase domain (Fig. 1e–g and Supplementary Fig. 7). The conserved hydrophobic residues Ile872, Leu875, Leu877, Ile880 and Phe881 were previously identified as being important for INCENP binding to AURKB αC^15^. All of these residues bind to the top face of αC, while INCENP 887–901 binds against the bottom face. The aromatic stacking interaction between INCENP Trp897 and AURKC His97 and His190 is prominent and, combined with the extensive hydrogen bonding network around INCENP pS893 and pS894, ensures that both sides of αC and the activation loop are held in an active conformation.

### TSS motif phosphorylations contribute to kinase activation

**Fig. 2:**
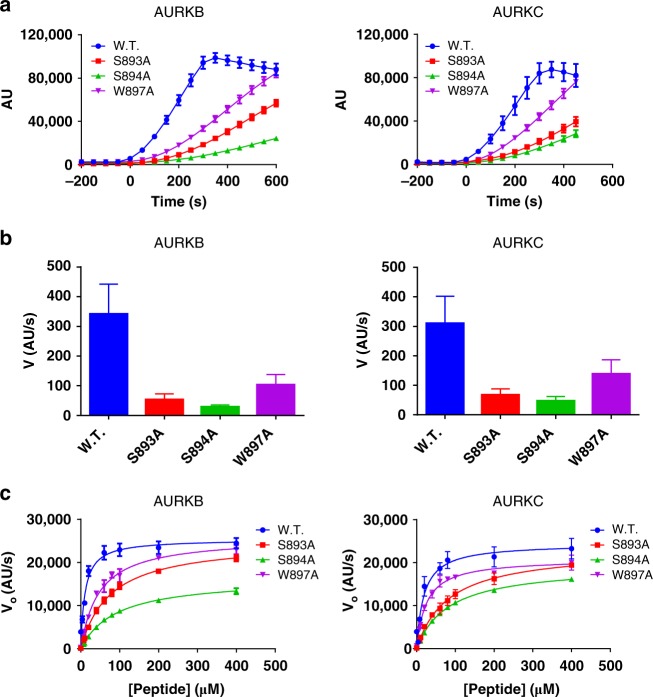
Enzymatic assays of AURKB or AURKC with wild-type or mutant INCENP. Mutation of the INCENP phosphorylation sites Ser893 or Ser894, or of INCENP Trp897 which is also important for Aurora kinase:INCENP binding, altered the enzymatic properties considerably. All measurements were made in triplicate. **a** Loss of TSS motif binding decreases the reaction rate for both AURKB and AURKC on a peptide substrate. **b** Quantification of the rate of reaction during the linear reaction phase of **a**. Error bars show standard error from the linear regression. **c** Measurement of initial rate of reaction (V_0_) at varying peptide substrate concentrations, with curves calculated by non-linear regression (curve-fitting) to the Michaelis–Menten equation. Calculated *K*_M_ and *K*_cat_ values from non-linear regression are shown in Table 2; the mutants show significant variation in *K*_M_ but similar *K*_cat_ values (with the possible exception of S894A)

After seeing that the TSS motif phosphorylations were involved both in stabilising the conformation of the activation loop and in stabilising helix αC, we speculated that lack of TSS motif phosphorylation would alter the substrate affinity/selectivity of the Aurora:INCENP complex. To test this hypothesis we overexpressed and purified AURKB:INCENP and AURKC:INCENP complexes containing TSS motif mutations S893A or S894A which prevent phosphorylation. We also tested W897A; Trp897 is adjacent to the TSS motif and forms substantial binding interactions with AURKC, in particular an aromatic stacking “sandwich” between His90 and His197 (Fig. 1e) and we reasoned that W897A might also destabilise the active conformation of INCENP bound to AURKB or AURKC. These complexes were used in real-time enzymatic assays to measure *K*_M_ for a generic peptide substrate. All three mutations resulted in a reduced rate of reaction with peptide substrate (Fig. 2a, b), as well as a reduced rate of auto-phosphorylation when in the absence of a peptide substrate (Supplementary Fig. 8). Analysis of the initial rates of reaction by non-linear regression to a Michaelis–Menten model allowed calculation of the *K*_M_(substrate) and *K*_cat_ values for the reaction (Fig. 2c, d); while the *K*_cat_ values were largely similar except for S894A (which is the most buried of the mutated residues and has the most hydrogen bonds), the *K*_M_ was in all cases significantly increased by the lack of TSS motif phosphorylation. This leads to the conclusion that INCENP TSS motif phosphorylation changes the substrate selectivity of the AURKB or AURKC complexes, as expected for a mechanism in which phosphorylated INCENP stabilises the activation loop and these two motifs together provide the binding site for the substrate.

**Table 2:** *K*_M_ and *K*_cat_ values from non-linear regression of the data in Fig. 2c

	INCENP	*K*_cat_ (10^3^ s^−1^)	*K*_m_(peptide) (μM)
AURKB	w.t.	50.8 ± 1.5	10.8 ± 1.5
	S893A	50.1 ± 0.9	75.5 ± 3.6
	S894A	32.9 ± 0.7	90.6 ± 4.7
	W897A	51.9 ± 1.2	47.1 ± 3.2
AURKC	w.t.	48.8 ± 2.3	18.7 ± 3.6
	S893A	46.3 ± 1.4	83.0 ± 6.1
	S894A	38.8 ± 0.6	83.0 ± 3.1
	W897A	41.8 ± 0.9	25.1 ± 1.9

*Note*: The data shown are the best-fit values ± standard error

### TSS motif phosphorylation stabilises Aurora:INCENP

**Fig. 3:**
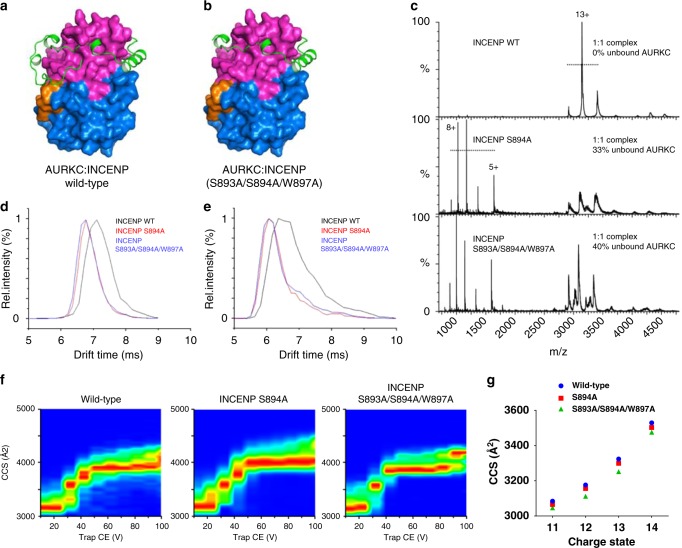
TSS motif binding affects AURKC:INCENP binding and shape. **a**, **b** Models of the effect of loss of TSS motif binding from a phosphorylated and ordered INCENP TSS motif as seen in the crystal structure (**a**) to an unphosphorylated TSS motif and disordered C-terminal part of INCENP (**b**). **c** Native mass spectra of AURKC:INCENP complexes show that under the same gentle ionisation conditions the wild-type AURKC:INCENP complex remains intact while the non-phosphorylated INCENP mutants are only partially bound. This is shown by the single peak series around *m*/*z* 3000–3200 for the wild-type AURKC:INCENP complex (MW expected 40501 Da, observed 40747 Da; one AURKC phosphorylation and two INCENP phosphorylations), while there are separate charge state series for the individual AURKC and INCENP proteins when samples of INCENP mutant complexes were measured (AURKC:INCENP-S894A MW expected 40059 Da, observed 40224 Da; one AURKC phosphorylation and one INCENP phosphorylation, and AURKC:INCENP-S893A/S894A/W897A MW expected 39928 Da, observed 40009 Da; one AURKC phosphorylation). **d**, **e** Without a phosphorylated INCENP TSS motif the AURKC:INCENP complexes have shorter ion mobility drift times (12+ and 13+ charge states shown). **f** Collision-induced unfolding shows the AURKC:INCENP mutant complexes reach the greatest extension already at lower energies consistent with greater structural flexibility. **g** The experimental CCS values of the AURKC:INCENP complexes were calculated after calibration from the ion mobility drift times. At all charge states the INCENP mutant complexes have a reduced collision cross-section consistent with a reduced size of the compact, ordered and folded protein domain

Structure comparison with partially active AURKB:INCENP reveals the extent of the disorder-order transition on moving to the active conformation (Supplementary Fig. 7). Since the active conformation has a phosphorylated kinase activation loop, as well as the phosphorylated INCENP TSS motif we wanted to verify that the INCENP TSS motif phosphorylations themselves make a significant contribution to this transition. We prepared complexes of AURKC:INCENP, AURKC:INCENP-S894A and AURKC:INCENP-S893A/S894A/W897A. S894A would be expected to disrupt the majority of the hydrogen bonding around the phosphorylated TSS motif while the triple-mutant complex was prepared to give the maximum chance of distinguishing the effect of the TSS motif. We hypothesised that the mutant complexes would have greater structural flexibility in the region of the TSS motif, which would be less strongly bound to AURKC. The mutant complexes would therefore have a different average shape and cross-sectional area compared to wild-type (Fig. 3a, b).

We utilised native electrospray ionisation (ESI) with ion mobility-mass spectrometry (IM-MS) to measure the degree of complex formation (% bound, at a concentration of ca. 10 µM), collision cross sections (rotationally averaged sizes, CCS) and intrinsic stabilities (gas-phase collision-induced unfolding profiles) of these AURKC:INCENP complexes. First, native MS showed that, under the experimental conditions used, with the wild type INCENP virtually 100% AURKC:INCENP complex is formed, while each of the INCENP mutant samples contained significant unbound AURKC (Fig. 3c) and unbound INCENP despite the proteins being purified in the same way and the analysis in the mass spectrometer being done with the same energies. This is consistent with loss of the hydrogen bonds to the INCENP phosphoserines leading to a weaker AURKC:INCENP interaction for the mutants. Second, the INCENP mutants showed slightly but significantly reduced ion mobility drift times (Fig. 3d, e) and, consequently, reduced collisional cross-sections (Fig. 3g), implying that conformations of the ordered protein are on average more compact. Third, the mutants showed an altered gas-phase unfolding profile with some unfolding transitions occurring at lower collision energies in the triple mutant (Fig. 3f). While the single INCENP S894A mutation displayed an effect, S893A/S894A/W897A showed a larger change (Fig. 3g) consistent with a model in which binding of the phosphorylated TSS motif serine residues and Trp897 provide additional stability to the AURKC:INCENP complex.

To analyse the relative accessibility of the INCENP and AURKC phosphorylations, the rate of dephosphorylation was assessed by incubating AURKB:INCENP or AURKC:INCENP complexes with the bacteriophage lambda phosphatase and monitoring by ESI-MS. The data shows that INCENP dephosphorylation occurs at least simultaneously and likely before dephosphorylation of the kinase (Supplementary Fig. 9). Dephosphorylation of the complexes with INCENP mutations appeared marginally faster in all cases (two examples shown in Supplementary Fig. 10) but experimental variables including differing phosphatase specificity for the mutants cannot be ruled out.

### VX-680 partially disrupts activation loop and TSS motif

**Fig. 4:**
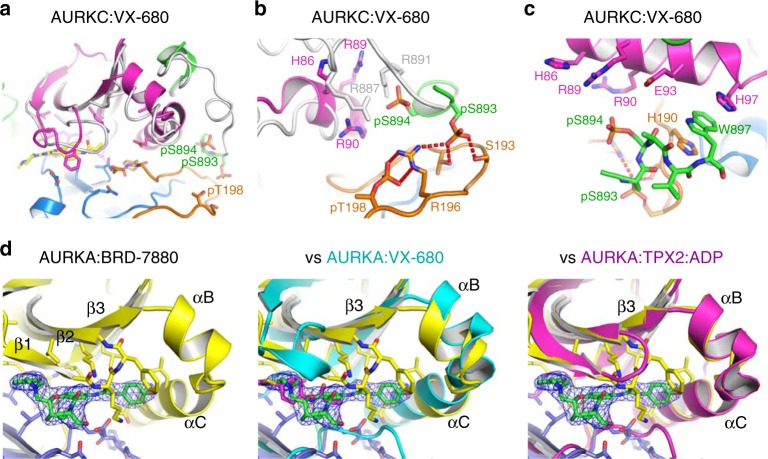
VX-680 binding partially disrupts AURKC:INCENP and affects the substrate binding groove. **a** Crystal structure of AURKC:INCENP:VX-680. AURKC is in magenta for the N-lobe, blue for the C-lobe and orange for the activation loop. INCENP is shown in green, VX-680 in yellow. The parts of the AURKC:INCENP:BRD-7880 structure that differ in position from the structure with VX-680 are shown in grey, illustrating the parts of AURKC:INCENP disrupted by VX-680 binding. **b** VX-680 binding to AURKC disrupts INCENP binding to the activation loop, disrupting the substrate binding surface. INCENP is shown in green and the part of INCENP not observed in the crystal structure is shown in grey, based on the structure of AURKC:INCENP:BRD-7880. **c** Binding of INCENP Trp897 to the C-terminal part of AURKC αC is maintained in the presence of VX-680. **d** A crystal structure of AURKA with BRD-7880 shows that the binding mode of BRD-7880 is conserved. Comparison of this structure with previously published structures of AURKA with VX-680 shows that, as with AURKC, BRD-7880 allows an Aurora kinase conformation resembling the active form exemplified by AURKA:TPX2:ADP, while VX-680 causes a rotation of the N-lobe that disrupts the positions of αB and αC. A 2*F*_o_–*F*_c_ electron density map is shown in blue around BRD-7880, contoured at 1.0 σ (0.14 eÅ^−3^)

To provide additional support that the observed conformations of INCENP and AURKC in the crystal structure were not due to crystal packing considerations, we also co-crystallised the activated AURKC:INCENP complex with the pan-Aurora kinase inhibitor VX-680 (tozasertib) (Fig. 4a). Crystals of AURKC:INCENP bound to VX-680 grew under different conditions to those with BRD-7880 and were in a different crystal form (Table 1). The best crystal diffracted to 2.25 Å resolution and allowed determination of the structure by molecular replacement using the coordinates of AURKC from the BRD-7880 co-crystal structure. In the final model INCENP binding and the activation loop conformation are conserved (Supplementary Fig. 7), but binding of the region of INCENP containing the TSS motif to AURKC is partly disrupted. The TSS phosphorylations are clearly bound to AURKC in the same conformation as seen in the BRD-7880 co-crystal structure (Fig. 4b), confirming the binding mechanism, however this binding is weaker with disrupted hydrogen bonding from pThr198 to Arg90, and Arg887 to pSer894, while the critical INCENP residues for binding pS894, Arg887 and Arg891, are disordered. The binding of INCENP Trp897 to the C-terminal end of AURKC αC is maintained (Fig. 4c).

These observations suggested that either the new crystal form or the presence of VX-680 disrupts the proper conformation of the N-terminal part of αC and αB, preventing proper binding of INCENP to form the activated conformation. Comparison of the AURKC:INCENP structure with VX-680 to the structure with BRD-7880 revealed a rotation of the N-lobe relative to the C-lobe as also seen in the comparison of AURKA:TPX2:ADP and AURKA:TPX2:VX-680 (Fig. 4d); this rotation results in a movement of αC and especially αB, and is presumed to be responsible for the partial disorder of INCENP relative to the structure with BRD-7880. Overall, the AURKC:INCENP:VX-680 structure superimposes with AURKB:INCENP:VX-680 better than does the AURKC:INCENP:BRD-7880 structure, with a r.m.s.d. of 0.67 Å over 172 Cα AURKC atoms compared to 0.80 Å over 177 Cα atoms for the BRD-7880 structure which fits with the model of conformational change induced by VX-680.

### Analysis of BRD-7880 binding and AURKA/AURKB selectivity

**Fig. 5:**
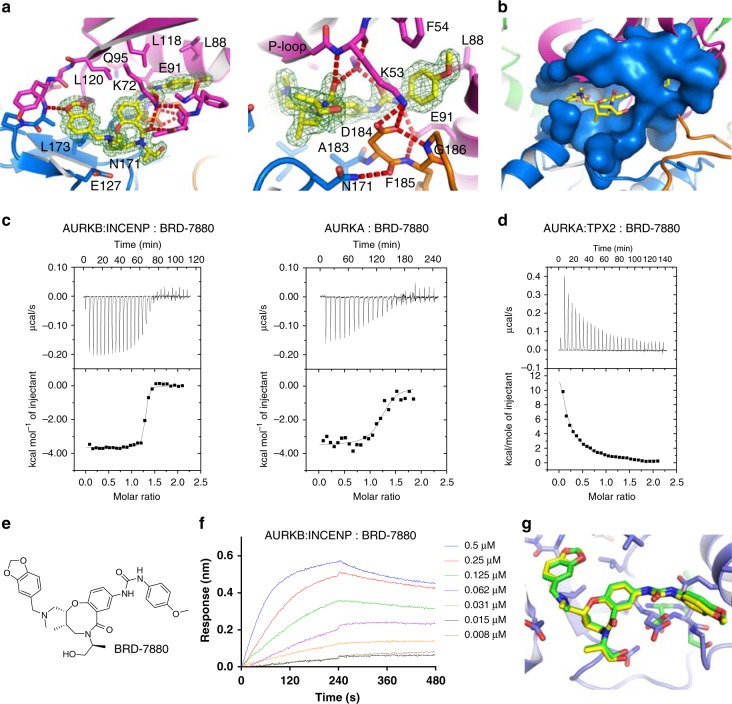
BRD-7880 binding to AURKC:INCENP. **a** BRD-7880 forms a single hydrogen bond to the kinase hinge region from its benzo−1,3-dioxole moiety, and multiple hydrogen bonds to the P-loop (Lys53) and to Lys72 and Glu91. A 2*F*_o_-*F*_c_ electron density map is shown in green around BRD-7880, contoured at 1.0 σ (0.4 eÅ^-3^) **b** The central 8-membered ring of BRD-7880 allows excellent shape complementarity to the ATP-binding site of AURKC. **c** Isothermal titration calorimetry confirmed BRD-7880 has significantly higher affinity for AURKB:INCENP over AURKA, due to more favourable entropy of binding (Supplementary Table 2). **d** Selectivity of BRD-7880 for AURKB:INCENP over AURKA:TPX2 is increased due to a non-ideal interaction, most likely the forced, possibly partial, dissociation of TPX2 from AURKA upon BRD-7880 binding. **e** Chemical structure of BRD-7880. **f** BRD-7880 has slow binding kinetics with AURKB:INCENP, with the long half-life of dissociation contributing to its potent binding. **g** BRD-7880 binds to AURKC:INCENP and AURKA in the same conformation, with the active site residues of AURKA adopting the same conformations as those of AURKC, with the exception of the DFG motif (residues 274 to 276 of AURKA). AURKC is shown in blue with bound BRD-7880 in yellow, and AURKA is shown in green

BRD-7880 was identified as a potent and specific AURKB inhibitor with a similar activity profile against cancer cell lines as VX-680 and *K*_i_ values against AURKB and AURKA of 4 nM and 1570 nM, respectively^16^. The co-crystal structure with AURKC:INCENP reveals a completely ordered BRD-7880 molecule (Fig. 5a) which binds in the ATP-binding site. The benzo-1,3-dioxole moiety (Fig. 5e) forms a hydrogen bond to Tyr122 on the kinase hinge region, the central 8-membered ring forms a perfect shape complementary binding over Leu173 and Ala183, the urea motif binds over the DFG motif, while the terminal methoxyphenyl moiety binds against the αC helix in particular Leu88 and Glu91. The binding of the methoxyphenyl in front of Glu91 pushes Glu91 back towards Gln95 (Fig. 5a), closing the pocket between Glu91 and Gln95 often seen in previous structures of AURKB with inhibitors^14,19,20^ (and as seen with our AURKC:INCENP:VX-680 structure).

Interestingly the urea motif is inserted between, and forms hydrogen bonds to both of, Lys72 and Glu91, which would normally form a salt bridge in an active conformation. Lys72 also forms a hydrogen bond to the oxygen of the inhibitor’s cyclic amide and to the backbone of the glycine-rich loop at Lys53. Lys53 forms hydrogen bonds with Asp184 of the DFG motif, which is linked via a hydrogen bond to the backbone nitrogen of Gly186. Thus, there is a comprehensive set of hydrogen bonds joining BRD-7880, the glycine-rich loop, the DFG motif and Glu91 from the αC helix. The eight-membered ring at the core of BRD-7880 allows it to have excellent shape-complementarity with AURKC, while the hydrogen bonding between Lys53 and Asp184 partially encloses BRD-7880 within the binding pocket (Fig. 5b).

For further assessment of the selectivity of BRD-7880 for AURKB/AURKC over AURKA, and in particular to examine the effect on selectivity or potency caused by INCENP binding to AURKB or TPX2 binding to AURKA, we conducted biophysical interaction studies. Isothermal Titration Calorimetry (ITC) provided *K*_D_ values of 16 nM and 341 nM for BRD-7880 binding to AURKB:INCENP or AURKA (no TPX2), respectively (Fig. 5c, Supplementary Table 2). The greater affinity of BRD-7880 for AURKB is due to a more favourable entropy term, with the enthalpy of binding being very similar to that for AURKA as expected from the conservation of active site residues between AURKB and AURKA.

We also attempted to measure the binding of BRD-7880 to AURKA:TPX2 which would provide a truer comparison of the selectivity of BRD-7880. However, injection of pre-prepared AURKA:TPX2 complex into BRD-7880 solution was endothermic, with a very low N (stoichiometry) value (Fig. 5d). Repetition of this experiment in a high (500 mM NaCl) salt buffer produced still larger endothermic peaks, while a control injection of AURKA:TPX2 into buffer produced very small endothermic peaks (Supplementary Fig. 11). One possible explanation is that BRD-7880 causes a loss of some of the AURKA:TPX2 binding interactions; in this situation the ITC experiment measures two binding events, one exothermic and one endothermic, and we have not attempted to derive a *K*_D_ value for the AURKA:TPX2:BRD-7880 interaction except to observe that the binding of BRD-7880 to AURKA is much weaker in the presence of TPX2.

Binding of TPX2 to AURKA was previously shown to increase affinity for both ATP and a substrate peptide^21^, while the *K*_M_(ATP) of AURKA is substantially decreased in the presence of TPX2 (1.7 µM vs. 27 µM)^22^. The EC_50_ for activation of AURKA by TPX2 was measured as 2.3 nM^21^. However, TPX2 binding to AURKA decreased affinity of AURKA for two out of three inhibitors tested, including VX-680^21^. The single inhibitor whose IC_50_ against AURKA was not affected by TPX2 was one that is not predicted to interact with helix αC^21^. Thus, TPX2 favours binding of ATP and disfavours binding of inhibitors that disrupt an active conformation. Given that BRD-7880 does interact with αC, and breaks the important Lys-Glu interaction, the reduction in its binding affinity to AURKA in the presence of TPX2 is expected.

BioLayer Interferometry (BLI) measurements (Fig. 5f) revealed that BRD-7880 bound to AURKA:TPX2 with fast kinetics and to AURKB:INCENP with slow kinetics (*k*_a_ ~ 0.017 s^−1^µM^−1^, it was not possible to determine an accurate value for *k*_d_ due to the slow off-rate, but conservatively t_1/2_ >2000 s for dissociation). A crystal structure of BRD-7880 bound to AURKA (in the absence of TPX2) confirms that the binding mode of BRD-7880 is identical to that when bound to AURKC (Figs. 4d, 5g, Table 1), including that the DFG motif of both AURKA and AURKC is in the DFG-in position, although the two motifs do not superimpose perfectly, likely due to the disordered activation loop in the AURKA:BRD-7880 structure.

Taken together, these data show that the selectivity for AURKB over AURKA seen in the enzymatic assays^16^ is due to favourable entropy of binding and a longer residence time with AURKB. The ITC and crystal structure data suggest that a major reason for the good selectivity of BRD-7880 is that it can bind to AURKB or AURKC without disturbing INCENP binding, but does not bind to an intact AURKA:TPX2 complex.

## Discussion

Previously it was shown that sub-stoichiometric INCENP is sufficient for AURKB activation by phosphorylation at Thr232^23^. This fits with a mechanism where INCENP pS893/pS894 alters the substrate selectivity of AURKB/AURKC towards itself, enabling auto-activation by phosphorylation *in trans*. The structure of partially active AURKB:INCENP contains an activation loop domain swap;^15^ this may be a mechanism for phosphorylation of non-consensus sites^18^, and it is possible that phosphorylation of the INCENP TSS motif by AURKB also proceeds *in trans*, while INCENP is bound to an interacting kinase domain. Interestingly however, the AURKB residues Met249 and His250 (isoform 1 numbering) which are important in the formation of the AURKB dimerisation interface^15^ are not conserved in AURKC, and indeed in all of our experiments AURKC:INCENP appeared monomeric.

Recent reports have presented data on the dynamic conformational equilibrium of the AURKA activation loop, and the change in this conformational ensemble during activation by phosphorylation and TPX2. The data suggest that the phosphorylated activation loop shifts to a more ordered state in solution only upon TPX2 binding, accompanied by a population shift towards a DFG-in state^24–26^, and that phosphorylation itself alters the structure of the DFG-in state rather than causing a population shift away from the DFG-out state^24^, and that TPX2 binding can override the effects of inhibitors on activation loop conformation^27^. The data so far on AURKB and AURKC suggest the possibility of a similar mechanism. With a phosphorylated activation loop, but without phosphorylated INCENP binding, the activation loop does not form all hydrogen bonds expected at its phosphothreonine and adopts a partially active conformation, whereas in the presence of phosphorylated INCENP the activation loop is fully engaged (Fig. 1b, Supplementary Fig. 6). Previously it was shown that TPX2 binding protects AURKA from activation loop dephosphorylation^17,21,28,29^, supporting a mechanism in which the AURKA activation loop is less flexible in the presence of TPX2. Although analysis of AURKB and AURKC dephosphorylation kinetics is complicated by the concomitant dephosphorylation of INCENP, the data is at least consistent with reduced flexibility of the AURKC activation loop and the INCENP TSS motif, when the TSS motif is phosphorylated.

While INCENP TSS motif phosphorylation occurs readily and to completion during co-expression with AURKB/C in bacteria, in human cells there are a variety of additional proteins effecting regulation of Aurora kinase activation. The data presented here shows that the TSS motif is best described as a TSSxxW motif, while additional conserved residues are involved in the activation mechanism.

Recently it was reported that INCENP is mono-methylated on Arg887 by PRMT1, and that this promotes mitosis of cancer cells^30^. Knockdown of PRMT1 reduced phosphorylation of histone H3 Ser10 (H3S10), a key substrate of AURKB in the mitotic process, and also the levels of phosphorylated AURKB Thr232, indicating that activation and activity of AURKB were both reduced. It was shown that methylation of Arg887 was essential for proper chromosomal segregation and cell division in A549 or HeLa cells^30^. Our structures show that Arg887 plays a key role binding to pS894 and also forming part of the substrate binding groove (Fig. 1b, g). Methylation of Arg887 would reduce the partial positive charge on its side chain, and reduce the strength of its interaction of pS894. This in turn would reduce the affinity of INCENP for AURKB/AURKC, as seen by Deng et al., although it may be the change in substrate selectivity or *K*_M_ that is more significant in terms of activation of AURKB or phosphorylation of H3S10.

McKenzie et al. showed that citron kinase (CIT) phosphorylates the INCENP TSS motif, increasing AURKB activity, while AURKB phosphorylated CIT on its coiled-coil domain, which localised CIT to the mitotic spindle^31^. This additional example of synergistic activation and localisation via phosphorylation at the INCENP TSS motif emphasises that there is still much to learn about the roles and regulation of Aurora kinases in mitotic cell division.

PKCε phosphorylates AURKB at Ser227 which alters the substrate selectivity of AURKB towards a particular set of substrates, and this switch is essential for the completion of cell division after the abscission checkpoint^32^. AURKB Ser227 is equivalent to AURKC Ser193 (Fig. 1d) and this phosphorylation site is on one of the activation loop serine residues that binds the phosphorylated TSS motif (Fig. 1b). Phosphorylation at AURKB Ser227 may compete with INCENP binding and change the conformation of the activation loop and INCENP in this region, thus changing the substrate selectivity. Interestingly, the only activation loop residue not conserved between AURKB and AURKC is AURKC Thr191 which is an alanine in AURKB. Therefore the binding of INCENP pS893 is likely to be less strong to AURKB than AURKC, and therefore AURKB Ser227 correspondingly more available for phosphorylation by PKCε.

Petsalaki et al. reported that phosphorylation of AURKB at Ser331 by CHEK1 is required for full AURKB activation and that this phosphorylation is present during mitosis^33^. Ser331 is in a highly conserved region of AURKB and AURKC, but it is located on the opposite site of AURKB/AURKC from the INCENP TSS motif, and so it must be a different mechanism of activation from that of the TSS motif analysed here. Petsalaki et al. also showed that Ser331 phosphorylation was required for TSS motif phosphorylation in cells. In vitro, as we and others have shown, the INCENP TSS motif is readily phosphorylated by AURKB/AURKC, so it may be that other cellular components, possibly in the CPC, are exerting a regulatory effect.

Interfering with INCENP TSS motif binding to AURKB or AURKC may be an interesting anti-mitotic strategy. An inhibitor acting in this way could potentially have pleiotropic effects of (i) reducing AURKB or AURKC activity, or (ii) altering substrate selectivity, or (iii) modifying the protein scaffolding in the CPC, and (iv) achieving all of these while not completely abolishing kinase activity. Given the complex role of the Aurora:INCENP interaction in regulating the spatiotemporal events in mitosis/meiosis, the potential cellular results of inhibition through such a mechanism are unclear. Such an inhibitor would have a very different profile from any Aurora kinase inhibitor that has been previously tried in the clinic. Finally, the conformational change induced by VX-680, and resultant weakening of INCENP binding, is illustrative of the changes in protein scaffolding that can be induced even by orthosteric kinase inhibitors. It is an open question to what extent differing effects of Aurora kinase inhibitors may be due to weakening of INCENP binding and/or alteration of substrate binding.

## Methods

### Cloning

DNA for various residue ranges of human Aurora kinase C or human Aurora kinase B were PCR amplified and subcloned into the first cloning site of an in-house pET-Duet based bi-cistronic vector pHTvAmp1-SGC (Supplementary Table 1). DNA for residues 835–903 of human INCENP (NP_001035784.1) was PCR amplified and subcloned into the second cloning site of the same plasmid. The resulting constructs expressed the desired protein complex with an N-terminal hexahistidine tag and TEV (tobacco etch virus) protease tag cleavage site for Aurora kinase B/C (extension MGSSHHHHHHSQDPENLYFQ*GANS where asterisk (*) represents the TEV protease digestion site), and an additional N-terminal methionine for INCENP. The constructs were verified by DNA sequencing. Primer sequences can be found in Supplementary Table 1.

The Aurora A expression plasmid which includes residues 122–403 of human Aurora A (NP_003591.2) with a TEV protease cleavable hexahistidine tag, and the TPX2 expression plasmid which includes residues 1–44 of human TPX2, were kind gifts from Prof. Richard Bayliss.

### Protein expression and purification

After testing protein expression levels of the constructs in small scale cultures, selected AURKB:INCENP and AURKC:INCENP complexes were purified on a larger scale by the same method: The constructs were transformed into BL21(DE3) cells (SGC) that contained the pRARE2 plasmid from commercial Rosetta2 cells. The resulting colonies were used to inoculate 50 mL of LB media containing 100 µg mL^−1^ ampicillin and 34 µg mL^-1^ chloramphenicol which was left shaking at 37 °C overnight. This culture was used to inoculate 1 L volumes of LB media containing 80 µg mL^-1^ ampicillin at a ratio of 10 mL culture to 1 L fresh media. The cultures were grown at 37 °C with shaking until an OD600 of 0.5 was reached. The temperature was reduced to 20 °C, and when the OD600 reached 0.7 isopropyl β-D-1-thiogalactopyranoside (IPTG) was added to a final concentration of 0.5 mM and the cultures were left overnight. Cells were harvested by centrifugation, re-suspended in Binding Buffer (50 mM HEPES pH 7.5, 500 mM NaCl, 20 mM imidazole, 5% glycerol, 0.5 mM tris(2-carboxyethyl)phosphine (TCEP)).

The re-suspended cells were lysed by sonication, polyethylenimine (PEI) was added to a final concentration of 0.15%, and the insoluble debris was removed by centrifugation. The supernatant was passed through a column of 5 mL Ni-Sepharose resin (GE Healthcare). The resin was washed with Binding Buffer containing increasing amounts of imidazole before elution with Binding Buffer containing 250 mM imidazole. TEV protease was added to the eluate, which was dialysed into 20 mM HEPES pH 7.5, 500 mM NaCl, 5% glycerol, 0.5 mM TCEP (GF Buffer) overnight at 4 °C. The protein complex was further purified by passing through a gravity column of 5 mL Ni-Sepharose. The flow-through was collected and the column was washed with GF Buffer containing 30, 60, 90, 120 and 250 mM imidazole. The fractions containing the pure protein complex were pooled, concentrated to 5 mL and injected on a S75 16/60 gel filtration column (GE Healthcare) pre-equilibrated into GF Buffer. Fractions containing the Aurora:INCENP complex were pooled and concentrated by ultrafiltration. Protein identities were confirmed by electrospray ionisation mass spectrometry (ESI-MS).

AURKA was expressed and purified similarly to AURKB:INCENP, and TPX2 was purified on GST-Sepharose resin (GE Healthcare) in a buffer consisting of 50 mM HEPES pH 7.5, 500 mM NaCl, 5% glycerol, 0.5 mM tris(2-carboxyethyl)phosphine (TCEP), with elution performed using the same buffer with the addition of 10 mM reduced glutathione.

### Mutagenesis

Mutations were introduced by PCR into the INCENP coding DNA sequence of the expression plasmids for the AURKB:INCENP or AURKC:INCENP complexes. As the mutations were close together it was possible to create single or multiple mutations within the same PCR primer. The mutated constructs were verified by DNA sequencing. Mutant protein complexes were produced from the modified plasmids using identical expression and purification procedures as for the wild-type protein. Protein identities were confirmed by ESI-MS.

### Crystallisation and data collection

Crystals were obtained using the sitting drop vapour diffusion method at 4 °C.

Crystals of AURKC:INCENP:BRD-7880 grew from a mixture of 50 nL protein (7.5 mg mL^−1^) and 100 nL of a well solution containing 0.2 M sodium chloride, 25% PEG3350 and 0.1 M HEPES pH 7.5. Crystals were equilibrated into reservoir solution plus 25% ethylene glycol before cryo-cooling in liquid nitrogen.

Crystals of AURKC:INCENP:VX-680 grew from a mixture of 100 nL protein (8.7 mg mL^−1^) and 50 nL of a well solution containing 20% PEG3350, 10% ethylene glycol, 0.1 M bis-tris-propane pH 7.5, 0.2 M sodium sulfate. Crystals were equilibrated into reservoir solution containing 25% ethylene glycol before freezing in liquid nitrogen.

Crystals of AURKA:BRD-7880 grew from a mixture of 100 nL protein (27 mg mL^−1^) and 50 nL of a well solution containing 0.2 M potassium citrate, 0.1 M Bis-Tris propane pH 7.5, 20% PEG 3350, 10% ethylene glycol. Crystals were equilibrated into reservoir solution containing 25% ethylene glycol before freezing in liquid nitrogen.

All data was collected at 100 K at the Diamond Synchrotron. Data collection statistics can be found in Table 1. Protein concentrations were measured by UV absorbance, using the calculated molecular weights and estimated extinction coefficients using a NanoDrop spectrophotometer (Thermo Scientific).

### Structure determination

The diffraction data was indexed and integrated using MOSFLM^34^ or XDS^35^, and scaled using AIMLESS^36^. The AURKC:INCENP:BRD-7880 structure was solved by molecular replacement using PHASER^37^ and the structure of human AURKB as a search model (PDB ID 4AF3^15^). There was one molecule of the AURKC:INCENP complex in the asymmetric unit. The model was built using Coot^38^ and refined with REFMAC5^39^. In the first cycle of model building 40 cycles of jelly-body refinement in REFMAC5 reduced the *R*_free_ from 0.52 to 0.45. After a subsequent cycle of deletion of atoms not matching the electron density (*R*_free_ = 0.44), where the INCENP coordinates matched those seen in the AURKB:INCENP structure these coordinates were added to the model. Rebuilding and refinement resulted in the final model.

The AURKC:INCENP:VX-680 structure was solved by molecular replacement using PHASER and the structure with BRD-7880 as a search model. Rebuilding and refinement in REFMAC5 resulted in the final model. The AURKA:BRD-7880 structure was solved by molecular replacement using PHASER and a previous structure of Aurora A (PDB ID 2J4Z^40^) as a search model. Rebuilding and refinement in REFMAC5 resulted in the final model. All models were validated using MOLPROBITY^41^.

### Enzymatic assays

The protein samples for enzymatic assays were all purified on the same day under identical conditions (Supplementary Fig. 12). Assays were run in black low volume 384-well plates in a total volume of 25 µL. Each well contained 10 µL kinase reaction (2.5× concentration), 5 µL ADP detection reagent A (5×) and 10 µL ADP detection reagent B (2.5×). Reagents A and B contained enzymes and co-factors that coupled ADP production to conversion of Amplex Red to Resorufin^42^. The final 1× assay buffer (in 25 µL) was 150 mM NaCl, 15 mM HEPES pH 7.5, 5 mM MgCl_2_, 0.1 mM EDTA, 0.1 mM EGTA. Triplicate measurements of 500 nM kinase:INCENP complex and varying peptide concentration were set up. ATP was injected to a final concentration of 20 µM after 10 measurement cycles. Measurements were made by fluorescence (excitation 530–10 nm, emission 590–20 nm) using a PheraStar FS plate reader (BMG Labtech).

For analysis of autophosphorylation the Aurora:INCENP complexes were at 500 nM and ATP was injected to a final concentration of 10 µM.

### BioLayer interferometry

The binding kinetics of BRD-7880 to the Aurora kinases was determined by the BLI method using an Octet RED384 system (FortéBio). All of the experiments were performed at 25 °C under buffer conditions of 20 mM HEPES pH 7.5, 500 mM NaCl, 0.5 mM TCEP. Constructs for biotinylated AURKA and biotinylated AURKB:INCENP were cloned, expressed and purified as previously. Biotinylation was confirmed by ESI-MS. The biotinylated proteins were immobilised onto Super Streptavidin biosensors, which were subsequently used in association and dissociation measurements each performed for a time window of 600 s. The interference patterns from the protein-coated biosensors with no compound and the uncoated biosensors with compound at corresponding concentrations were measured as two sets of controls. After double referencing corrections, the subtracted binding interference data were applied to the calculations of binding constants using the FortéBio analysis software provided with the instrument.

### Isothermal titration calorimetry

Measurements were made at 25 °C on a VP-ITC machine (GE Healthcare). All proteins were dialysed overnight into a buffer consisting of 50 mM HEPES pH 7.5, 500 mM NaCl, 5% glycerol, 0.5 mM TCEP. For AURKA and AURKB:INCENP the syringe was loaded with 0.2 mM protein and 28 × 10 μL injections were made into the cell, which was filled with 0.02 mM BRD-7880. For AURKA:TPX2 the syringe was loaded with 0.17 mM protein and 28 × 10 μL injections were made into the cell, which was filled with 0.017 mM BRD-7880. Data was analysed using MicroCal and Origin software.

### Native ion mobility mass spectrometry

The native ion mobility-mass spectrometry (IM-MS) measurements were performed on a Synapt G2 HDMS (Waters) under conditions which preserve native structure^43,44^. The samples were buffer exchanged into 250 mM ammonium acetate using Micro Bio-spin P6 columns (Bio-Rad), and sprayed at gentle instrument tuning conditions using nano-electrospray ionisation with home-made glass capillaries; spray capillary voltage 1.2 kV, sampling cone 30 V, trap CE 5.0 V, transfer CE 0.0 V, trap bias 45 V, nanoflow gas pressure 0.1 bar, backing pressure 3 mbar, source temperature 30 °C, IMS wave velocity 300 m/s, IMS wave height 35 V. The data were acquired and processed with Masslynx v4.1 software, and ion mobility drift times extracted using Driftscope v2.3 (both Waters). The collision cross sections (CCS) of the proteins were calibrated using known CCS values determined under native conditions as described previously^45^.

### Dephosphorylation assay

Protein samples of AURKB:INCENP or AURKC:INCENP were prepared at a final concentration of 1 mg mL^−1^ by dilution with 20 mM Hepes pH 7.5, 300 mM NaCl, 0.5 mM TCEP. MnCl_2_ was added to a final concentration of 1 mM and bacteriophage lambda phosphatase was added to a final concentration of 41 µg mL^−1^. Aliquots were removed periodically and immediately mixed with 0.1% formic acid before analysis by ESI-MS.

### Reporting summary

Further information on research design is available in the Nature Research Reporting Summary linked to this article.

## Supplementary information

Supplementary Information

Reporting Summary

Source Data

## Data Availability

Coordinates and structure factors have been deposited in the PDB under accession codes 6GR8, 6GR9 and 6GRA. The source data underlying Figs. 2a–c, Table 2 and Supplementary Fig. 8 are provided as a Source Data file. Other data are available from the corresponding author upon reasonable request.
