# Supplementary material for: Structural mechanism of synergistic activation of Aurora kinase B/C by phosphorylated INCENP
Source: Nat Commun. 2019 Jul 18;10:3166. doi: 10.1038/s41467-019-11085-0 (PMC6639382; doi:10.1038/s41467-019-11085-0)
Supplement: Supplementary file 1 — Supplementary Information [file 41467_2019_11085_MOESM1_ESM.pdf]

Supplementary Material

Structural mechanism of synergistic activation of Aurora kinase B/C by phosphorylated INCENP

K. R. Abdul Azeez et al.

Contents

Supplementary Table 1 – Protein Expression Constructs and Primer Sequences 3
Supplementary Figure 1 – Intact mass spectra of purified proteins.. 4
Supplementary Figure 2 – Electron density maps for phosphorylated residues............................ 6
Supplementary Figure 3 – Sequence alignment of human AURKB and AURKC isoforms 7
Supplementary Figure 4 – Sequence alignment of AURKB or AURKC from diverse organisms. 9
Supplementary Figure 5 – Sequence alignment of INCENP from diverse organisms................... 12
Supplementary Figure 6 – Comparison of activation loop conformations.................................. 13
Supplementary Figure 7 – INCENP binds in the same conformation to AURKB and AURKC but with additional ordered residues bound in the activated state 14
Supplementary Figure 8 – Rates of auto-phosphorylation of INCENP mutant AURKB or AURKC complexes.. 15
Supplementary Figure 9 – Dephosphorylation kinetics 16
Supplementary Figure 10 – Example dephosphorylation kinetics of INCENP mutants................ 18
Supplementary Table 2 – Isothermal Titration Calorimetry 20
Supplementary Figure 11 – Isothermal titration calorimetry of AURKA:TPX2 and BRD-7880 21
Supplementary Figure 12 – SDS-PAGE analysis of purified proteins used for enzymatic assays . 22
Supplementary References 23

Supplementary Table 1 – Protein Expression Constructs and Primer Sequences

Co-expression of AURKB (NP_004208.2) + INCENP 835-903 (NP_001035784.1)

Start Residue	End Residue	Good Soluble Protein Expression in E. coli?	Primer Sequences
Ser43	Ala344	YES	Forward: TAAAGAATTCGAGCCGCTCCAATGTCCA<br>Reverse: TATAAAGCTTTCAGGCGACAGATTGAAGGG
Ser43	Pro336	YES	Forward: TAAAGAATTCGAGCCGCTCCAATGTCCA<br>Reverse: TATAAAGCTTTCAAGGCAGCACCCTCCGA
Ser43	Arg332		Forward: TAAAGAATTCGAGCCGCTCCAATGTCCA<br>Reverse: TATAAAGCTTTCACCGAGAGTTGGCCCGG
Gln55	Ala344	YES	Forward: TAAAGAATTCGCAGAAGGTGATGGAGAATAGC<br>Reverse: TATAAAGCTTTCAGGCGACAGATTGAAGGG
Pro65	Ala344	YES	Forward: TAAAGAATTCGCCCGACATCTTAACGCG<br>Reverse: TATAAAGCTTTCAGGCGACAGATTGAAGGG
Arg70	Ala344	YES	Forward: TAAAGAATTCGCGGCACTTCACAATTGATG<br>Reverse: TATAAAGCTTTCAGGCGACAGATTGAAGGG

Co-expression of AURKC (NP_001015878.1) + INCENP 835-903 (NP_001035784.1)

Start Residue	End Residue	Good Soluble Protein Expression in E. coli?	Primer Sequences
Arg36	Ser309	YES	Forward: TAAAGAATTCGCGGCGCCTCACAGTCGA<br>Reverse: TATAAAGCTTTCAGGAAGCCATCTGAGCAC
Arg36	Ala305	YES	Forward: TAAAGAATTCGCGGCGCCTCACAGTCGA<br>Reverse: TATAAAGCTTTCAAGCACAGGGAGGCAGC
Arg36	Arg298	YES	Forward: TAAAGAATTCGCGGCGCCTCACAGTCGA<br>Reverse: TATAAAGCTTTCATCGGGAGTGGGCCTGA
Thr39	Ser309	YES	Forward: TAAAGAATTCGACAGTCGATGACTTTGAAATCGG<br>Reverse: TATAAAGCTTTCAGGAAGCCATCTGAGCAC
Thr39	Ala305	YES	Forward: TAAAGAATTCGACAGTCGATGACTTTGAAATCGG<br>Reverse: TATAAAGCTTTCAAGCACAGGGAGGCAGC
Thr39	Arg298		Forward: TAAAGAATTCGACAGTCGATGACTTTGAAATCGG<br>Reverse: TATAAAGCTTTCATCGGGAGTGGGCCTGA
Asp41	Ser309		Forward: TAAAGAATTCGGATGACTTTGAAATCGGGC<br>Reverse: TATAAAGCTTTCAGGAAGCCATCTGAGCAC
Asp41	Ala305	YES	Forward: TAAAGAATTCGGATGACTTTGAAATCGGGC<br>Reverse: TATAAAGCTTTCAAGCACAGGGAGGCAGC
Asp41	Arg298		Forward: TAAAGAATTCGGATGACTTTGAAATCGGGC<br>Reverse: TATAAAGCTTTCATCGGGAGTGGGCCTGA

INCENP Forward:	TAAACATATGGAGGCCCATCCCCGGAA
INCENP Reverse:	TATACTCGAGTCACTGCAGGGGCGGTGAG

Supplementary Figure 1 – Intact mass spectra of purified proteins

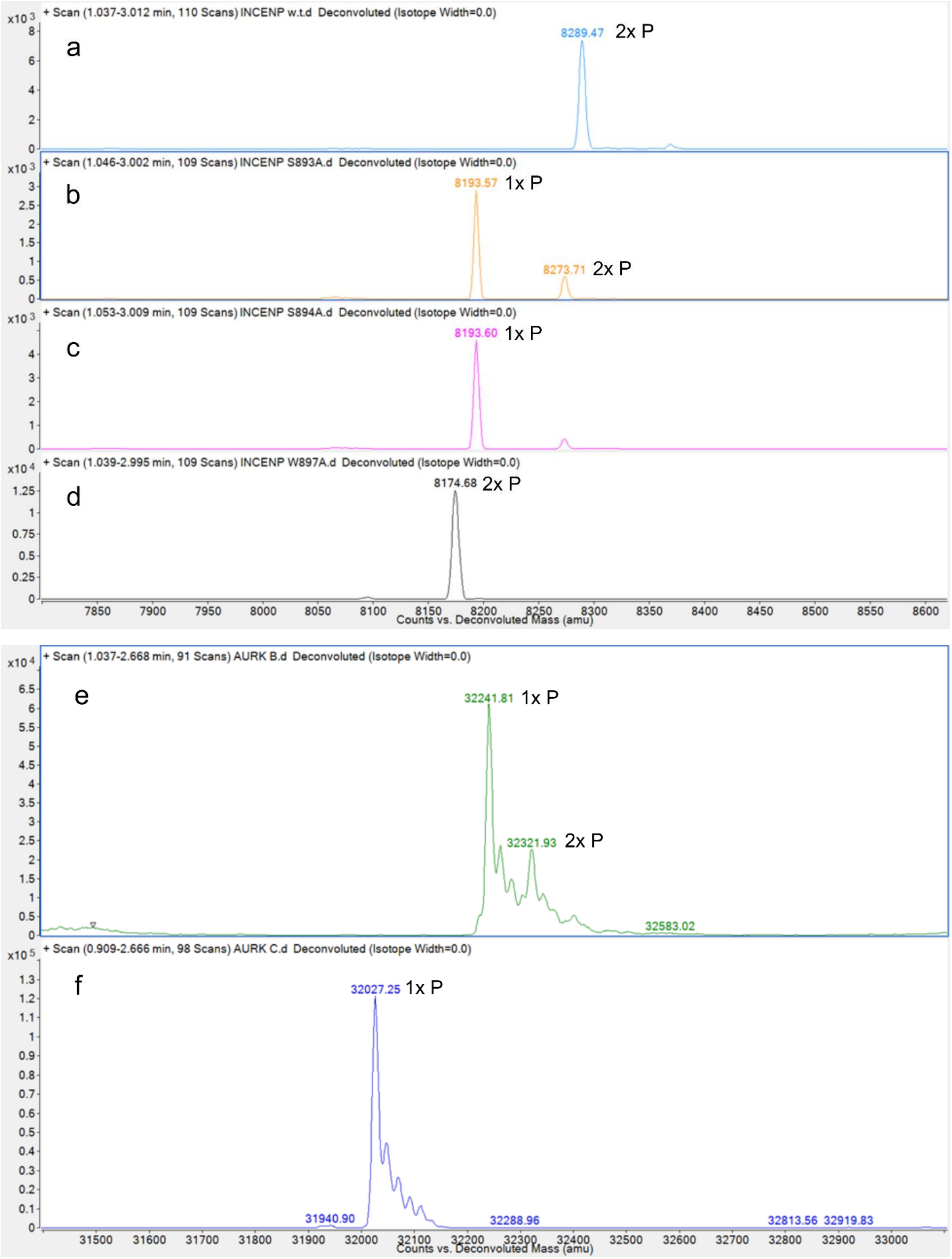

Example Intact mass spectra for **A.** INCENP wild-type, **B.** INCENP S893A, **C.** INCENP S894A, **D.** INCENP W897A, **E. and F.** AURKB and AURKC as used for enzymatic assays, the major peak in each spectrum represents a singly phosphorylated species (+80). The major peaks have been marked according to the number of phosphorylations on the protein. Note that the crystallised AURKC:INCENP protein contained an additional AURKC phosphorylation (Thr39) as seen in the structure (see Supplementary Figure 2), which may have been due to variation in expression conditions or because the crystallised AURKC construct was three residues longer on the N-terminus.

Supplementary Figure 2 – Electron density maps for phosphorylated residues

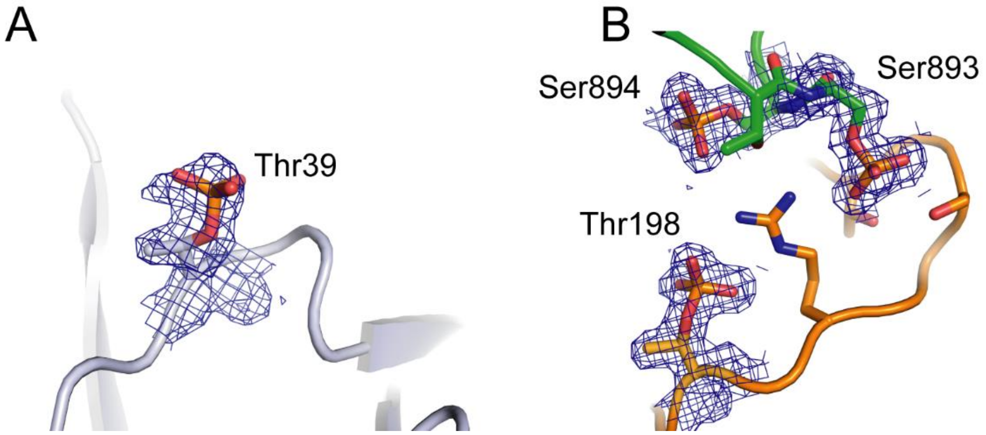

Electron density maps for (a) AURKC phospho-Thr39, (b) AURKC phospho-Thr198 and INCENP phospho-Ser893 and phospho-Ser894. A 2F_o-F_c electron density map is shown contoured at 1.0σ (0.4 eÅ^{-3}) only around the phosphorylated residues.

Supplementary Figure 3 – Sequence alignment of human AURKB and AURKC isoforms

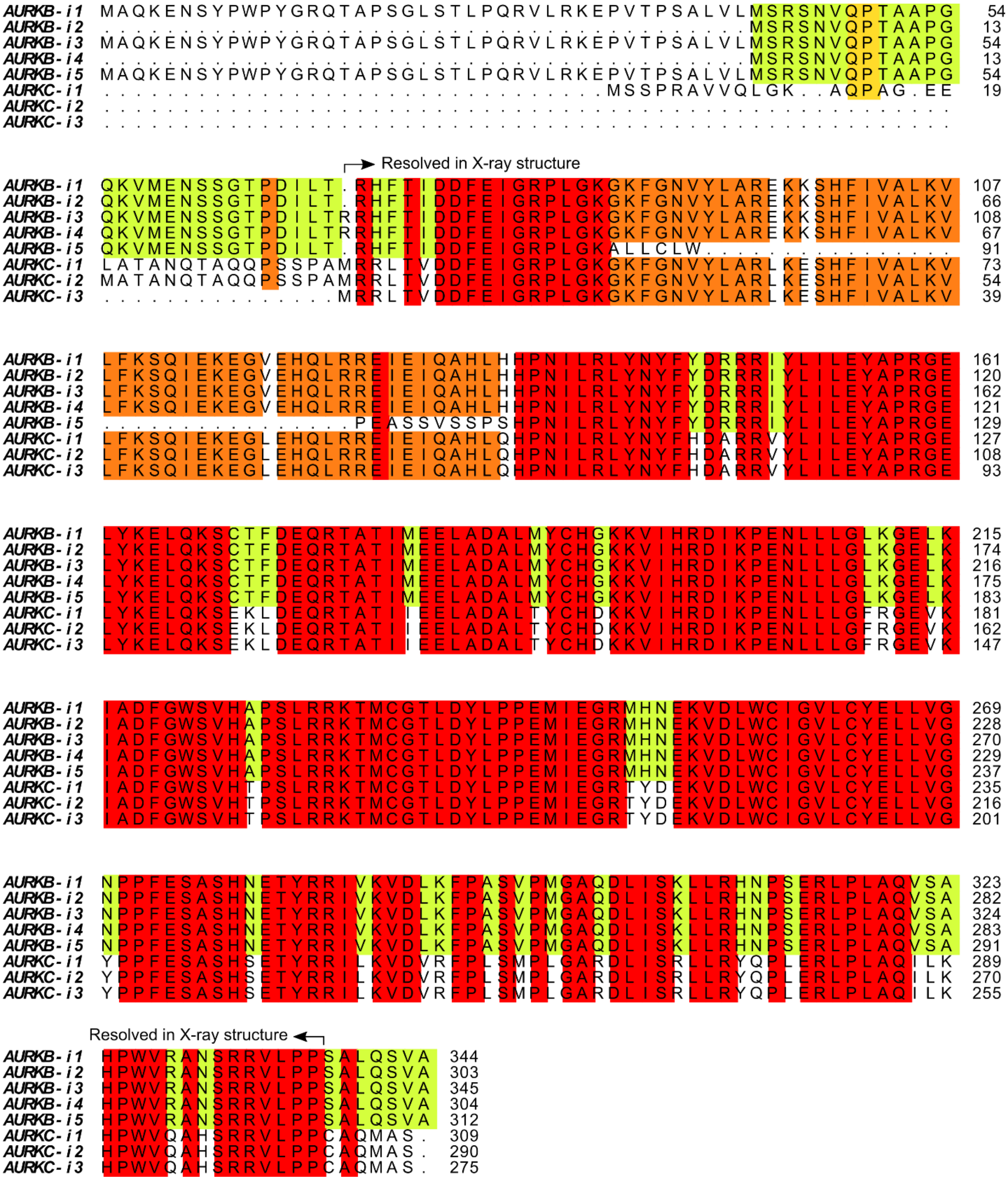

Sequence alignment of human AURKB and AURKC isoforms. The following sequences were used:

Gene	Isoform	NCBI reference

AURKB	1	NP_004208.2
AURKB	2	NP_001300880.1
AURKB	3	NP_001271455.1
AURKB	4	NP_001300881.1
AURKB	5	NP_001300882.1
AURKC	1	NP_001015878.1
AURKC	2	NP_001015879.1
AURKC	3	NP_003151.2

Two non-functional isoforms of AURKB were not included in the analysis.

The sequence alignment was created using ClustalO (1) and Aline (2).

Supplementary Figure 4 – Sequence alignment of AURKB or AURKC from diverse organisms.

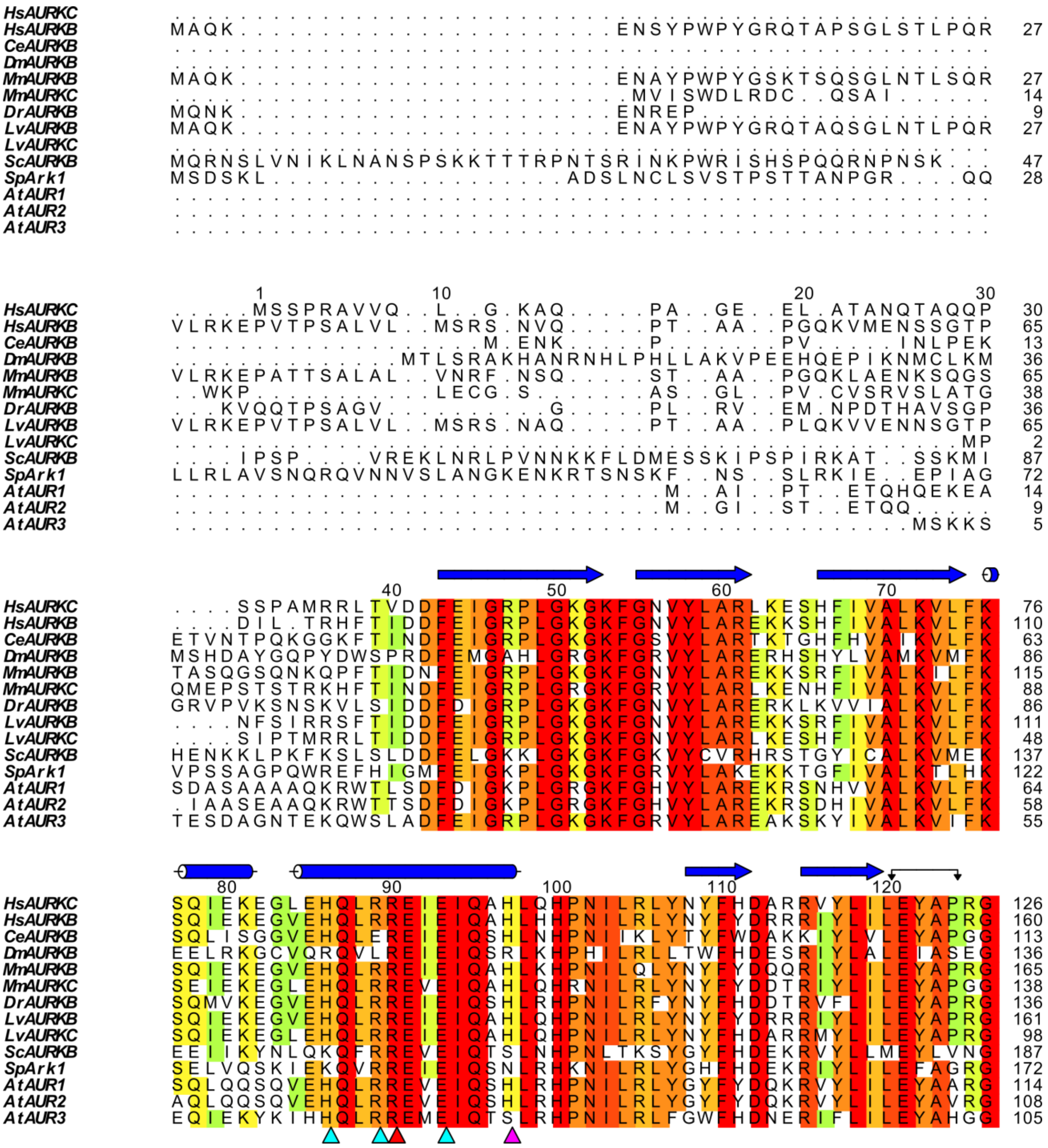

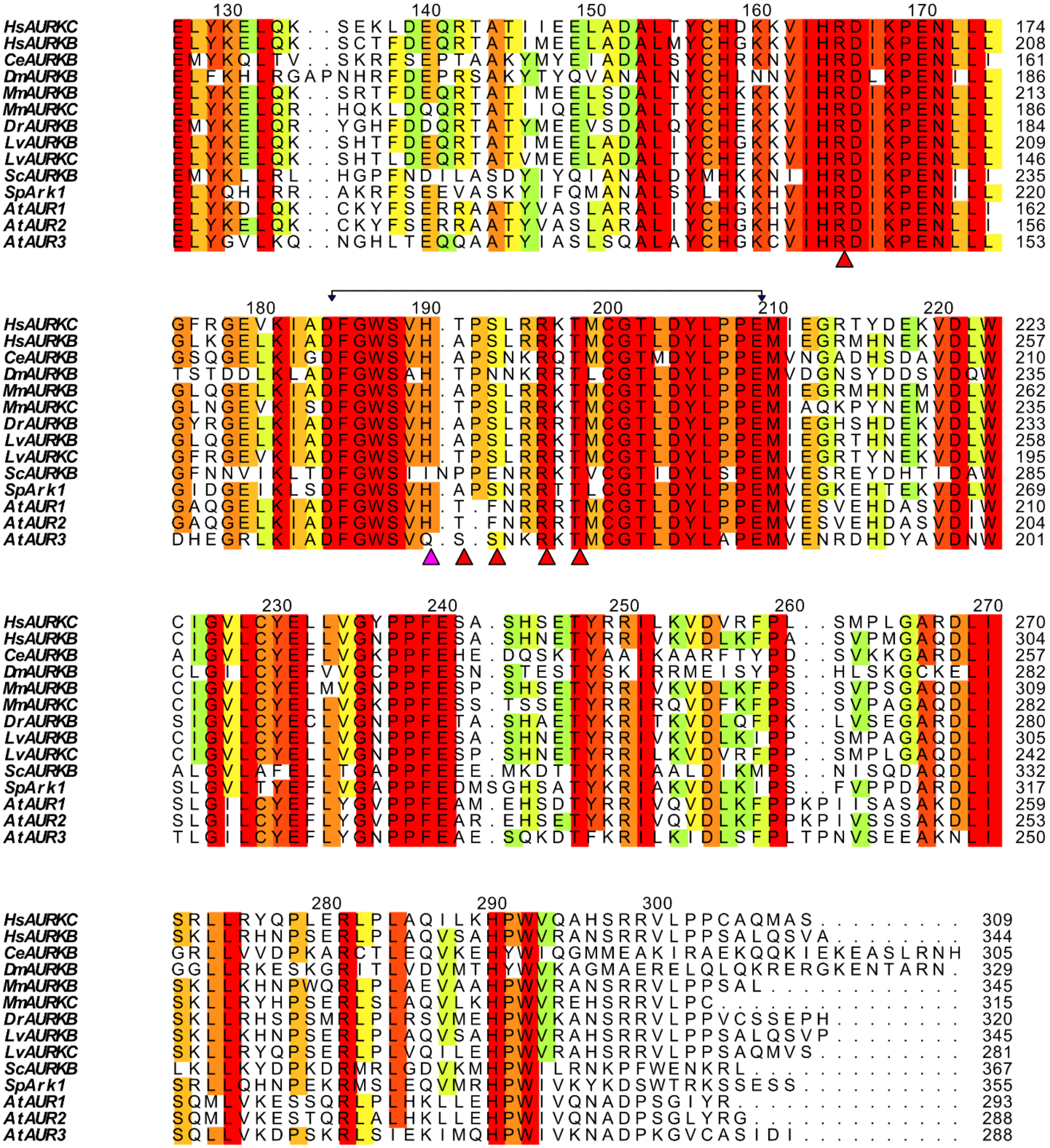

The sequence alignment was created using ClustalO (1) and Aline (2).

Residues involved in binding INCENP are marked with triangles below the alignment.

The following sequences were used:

Organism	Gene	NCBI Reference
Homo sapiens	AURKC	NP_001015878.1
Homo sapiens	AURKB	NP_004208.2
Caenorhabditis elegans	AURKB	NP_491714.1
Drosophila melanogaster	AURKB	NP_477336.1
Mus musculus	AURKB	NP_035626.1
Mus musculus	AURKC	NP_001074434.1
Danio rerio	AURKB	NP_997731.2
Lipotes vexillifer	AURKB	XP_007457875.1
Lipotes vexillifer	AURKC	XP_007457528.1
Saccharomyces cerevisiae S288c	AURKB	NP_015115.1
Schizosaccharomyces pombe 972h-	Ark1	NP_001018849.1
Arabidopsis thaliana	AUR1	NP_195009.1
Arabidopsis thaliana	AUR2	NP_180159.2
Arabidopsis thaliana	AUR2	NP_182073.1

Supplementary Figure 5 – Sequence alignment of INCENP from diverse organisms

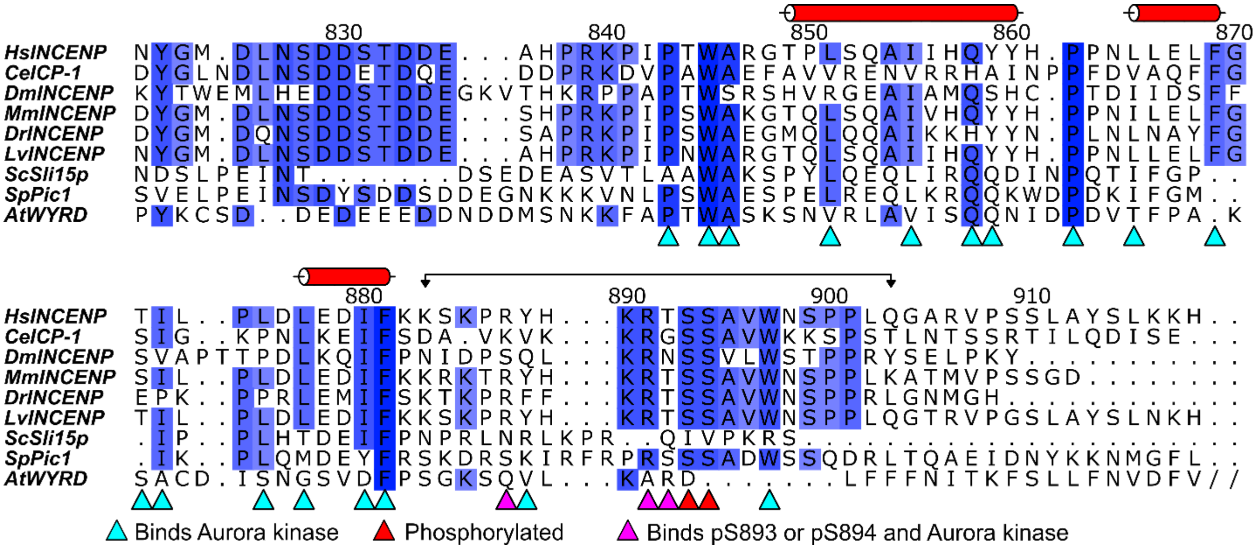

The sequence alignment was created using ClustalO (1) and Aline (2).

Residues involved in binding Aurora kinase are marked with triangles below the alignment.

The following sequences were used:

Organism	Gene	NCBI Reference
Homo sapiens	INCENP	NP_001035784.1
Caenorhabditis elegans	ICP-1	NP_490956.1
Drosophila melanogaster	INCENP	NP_523648.3
Mus musculus	INCENP	NP_057901.2
Danio rerio	INCENP	XP_009295991.2
Lipotes vexillifer	INCENP	XP_007462274.1
Saccharomyces cerevisiae S288c	Sli15p	NP_009714.3
Schizosaccharomyces pombe 972h-	Pic1	NP_596135.2
Arabidopsis thaliana	WYRD	NP_200393.1

Supplementary Figure 6 – Comparison of activation loop conformations

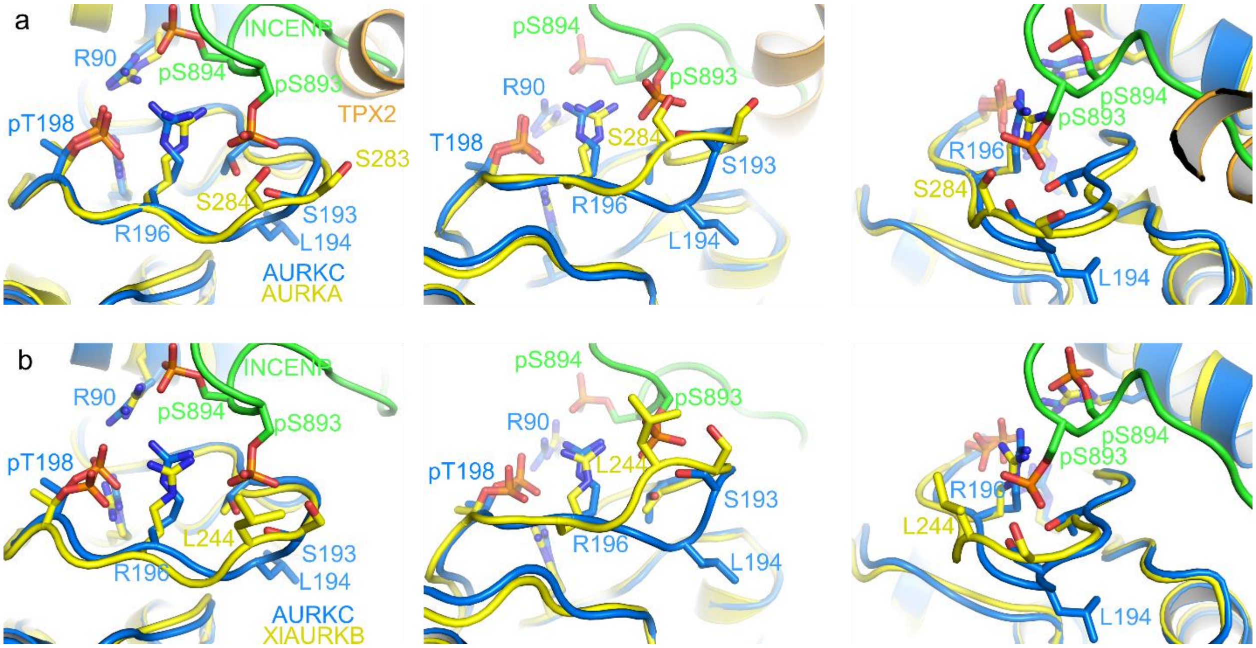

(a) The activation loop conformation of active-form AURKC:INCENP (in blue and green) is compared to active-form AURKA:TPX2 (in yellow and orange) (PDB ID 1OL5) (3). Three different viewing angles are shown in the three panels from left to right. **(b)** Active-form AURKC:INCENP (in blue and green) is compared to partially active Xenopus laevis AURKB:INCENP (in yellow and orange, lacking phosphorylated INCENP) (PDB ID 2BFY) (4). Three different viewing angles are shown in the three panels from left to right.

Supplementary Figure 7 – INCENP binds in the same conformation to AURKB and AURKC but with additional ordered residues bound in the activated state

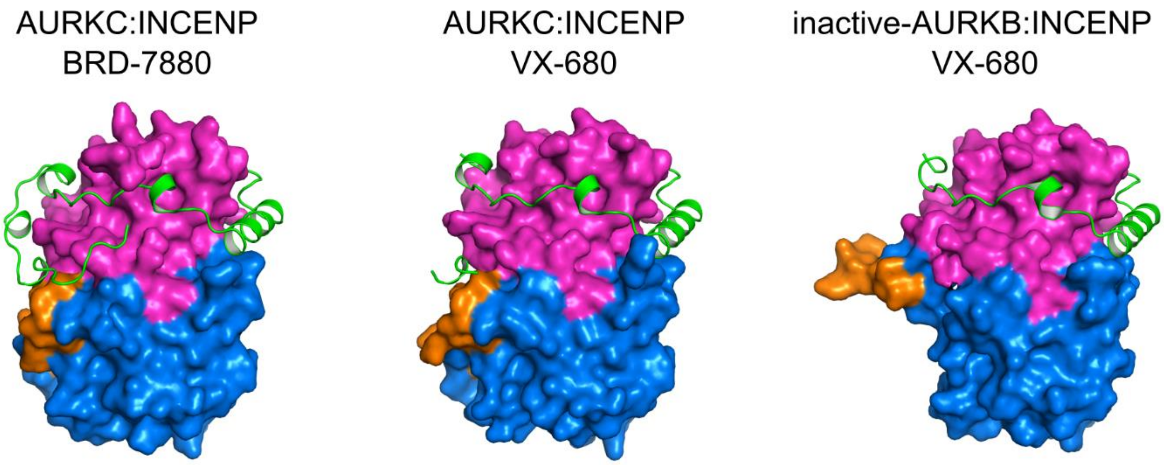

The two AURKC:INCENP structures obtained in this work are compared to the inactive structure of human AURKB:INCENP we previously published (5) (on the right, PDB ID 4AF3). The structures were superimposed and are viewed in the same orientation. The Aurora kinase N-lobes are in magenta, the C-lobes in blue, the activation loops in orange, and INCENP is coloured green.

Supplementary Figure 8 – Rates of auto-phosphorylation of INCENP mutant AURKB or AURKC complexes

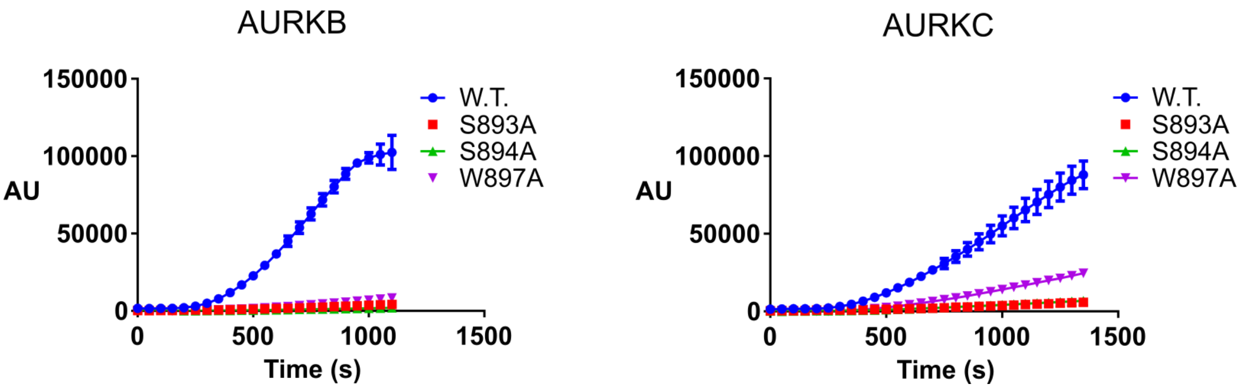

Auto-phosphorylation was monitored by detection of the ADP produced during the phosphorylation reaction in the absence of a peptide substrate.

Supplementary Figure 9 – Dephosphorylation kinetics

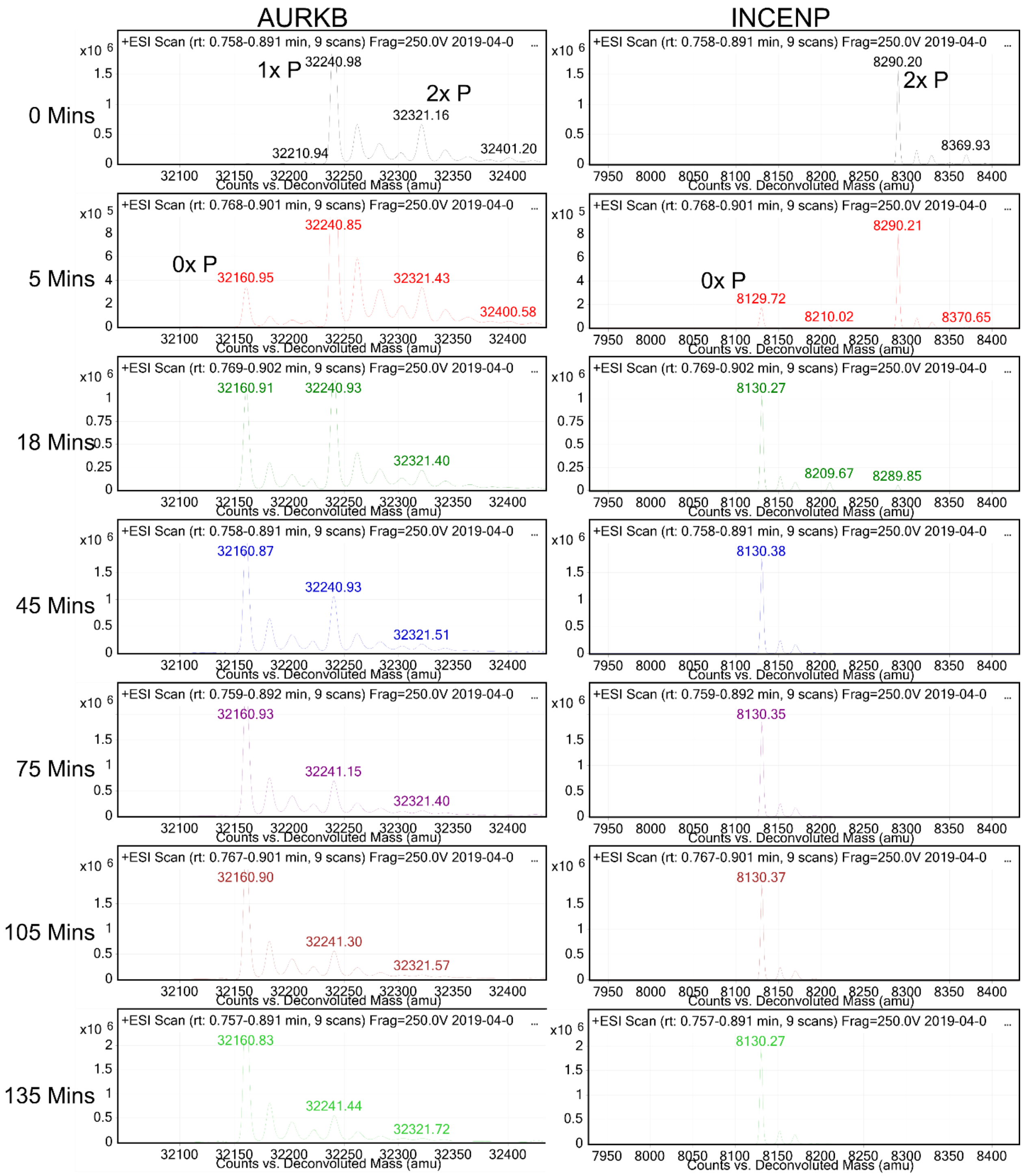

Time course of dephosphorylation of AURKB:INCENP.

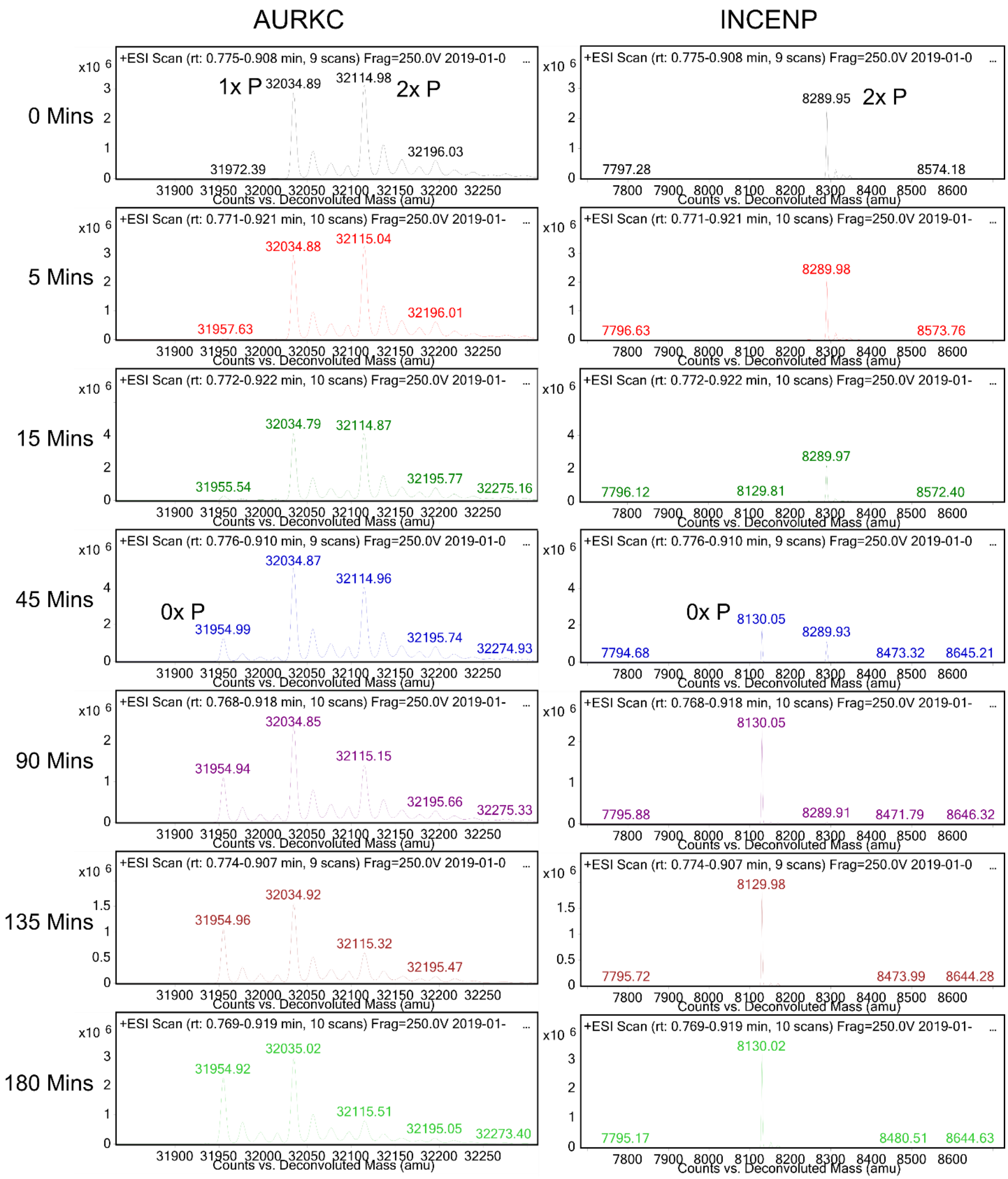

Time course of dephosphorylation of AURKC:INCENP.

Supplementary Figure 10 – Example dephosphorylation kinetics of INCENP mutants

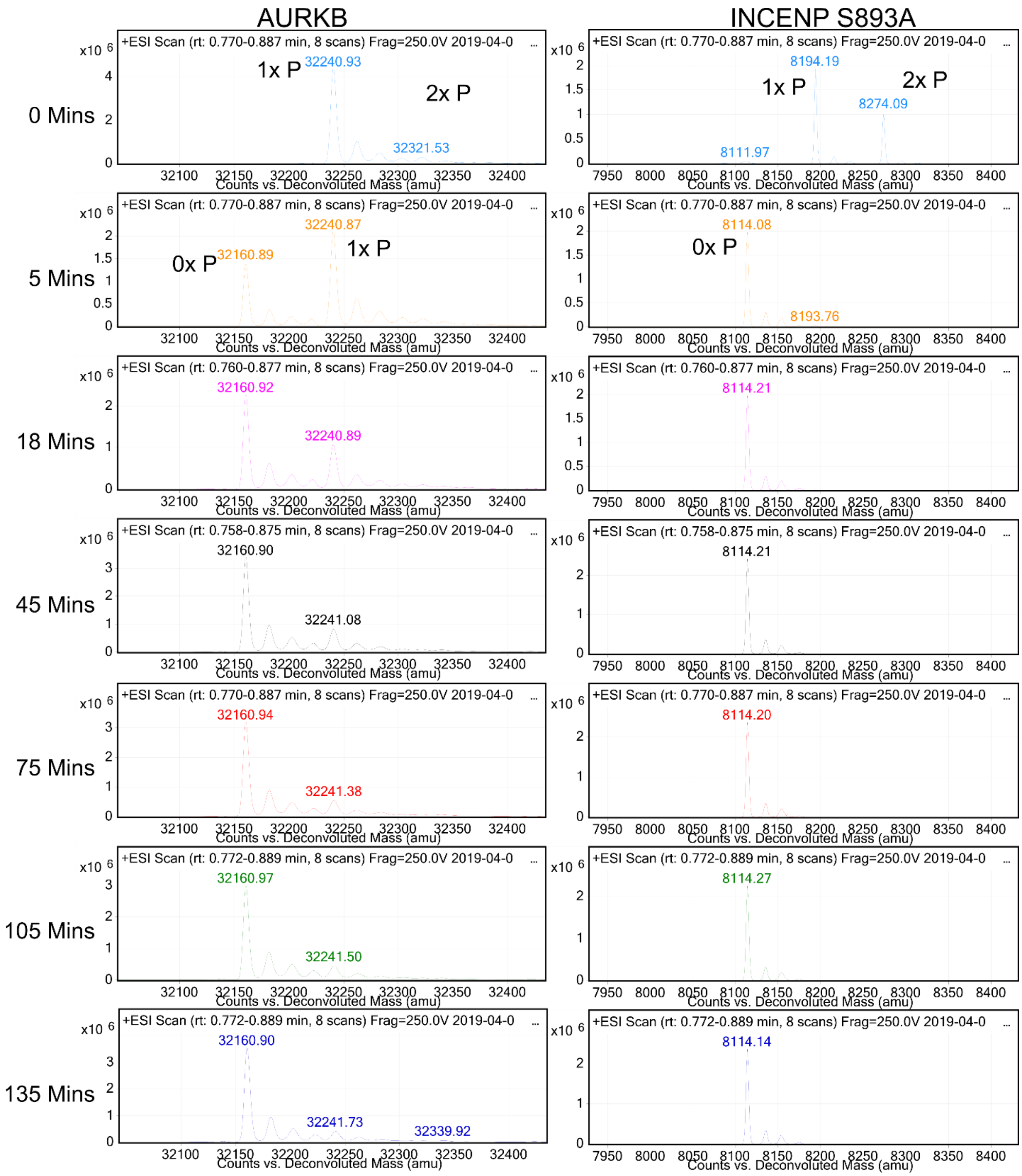

Time course of dephosphorylation of AURKB:INCENP S893A.

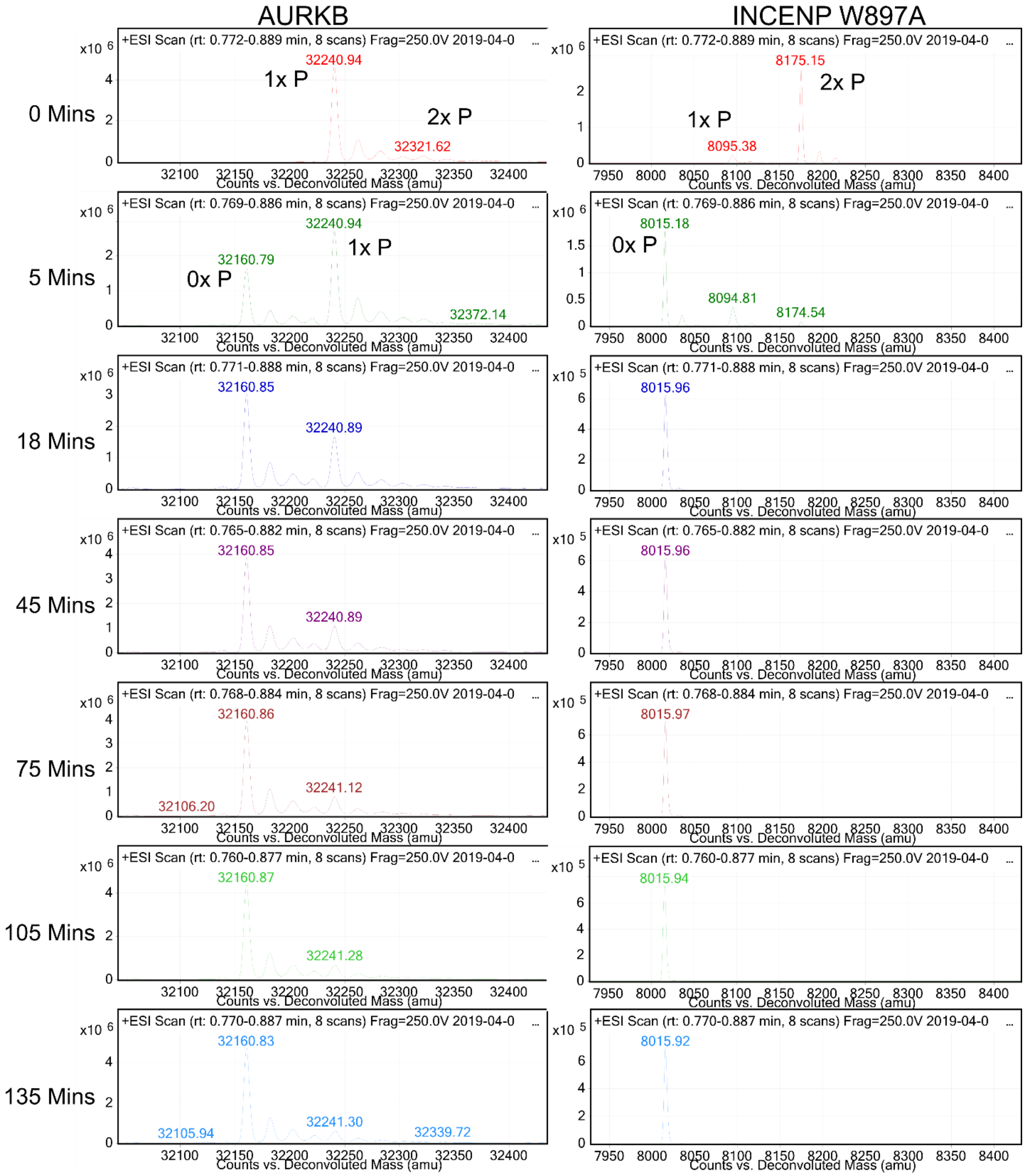

Time course of dephosphorylation of AURKB:INCENP W897A.

Supplementary Table 2 – Isothermal Titration Calorimetry

	**AURKB:INCENP BRD-7880**	**AURKA BRD-7880**	**AURKA:TPX2 BRD-7880**	**AURKA:TPX2 BRD-7880 (500 mM NaCl)**
Molar ratio (n)	1.26	1.23	**	**
ΔH (cal/mol)#	-3648 ± 26	-3507 ± 128	**	**
TΔS (cal/mol)	+6977	+5307	**	**
K_D (nM)#	16 ± 3.5	341 ± 160	**	**

Data were measured at 25 ^{o}C in 50 mM HEPES pH 7.5, 500 mM NaCl, 5% glycerol, 0.5 mM TCEP.

#Errors given in the table represent the error of the non-linear least squares fit to the experimental data.

** Values not calculated due to the inability to fit a single site model to the data correctly.

Supplementary Figure 11 – Isothermal titration calorimetry of AURKA:TPX2 and BRD-7880

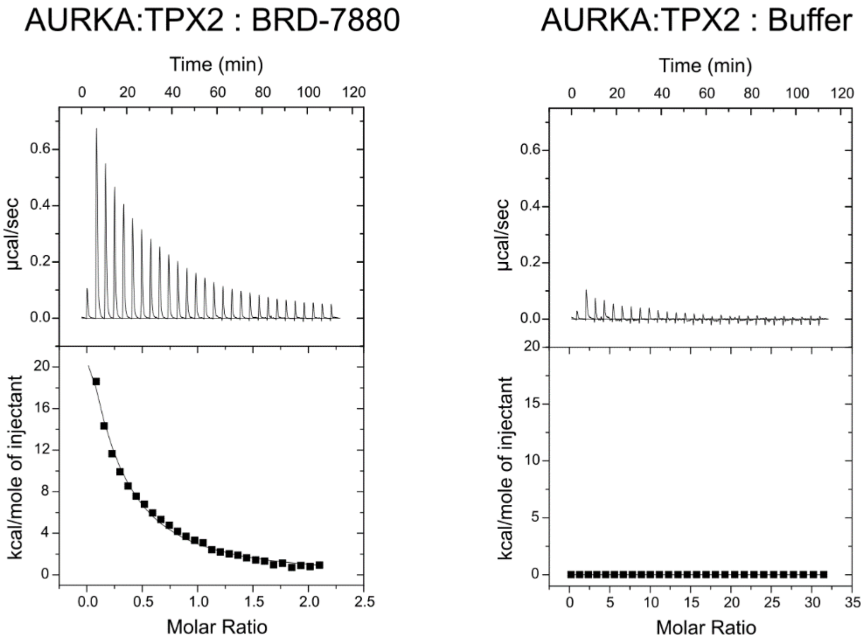

The injection of AURKA:TPX2 complex into a solution of BRD-7880 in buffer containing 500 mM NaCl (left) is compared to an injection of AURKA:TPX2 in buffer alone (right).

Supplementary Figure 12 – SDS-PAGE analysis of purified proteins used for enzymatic assays

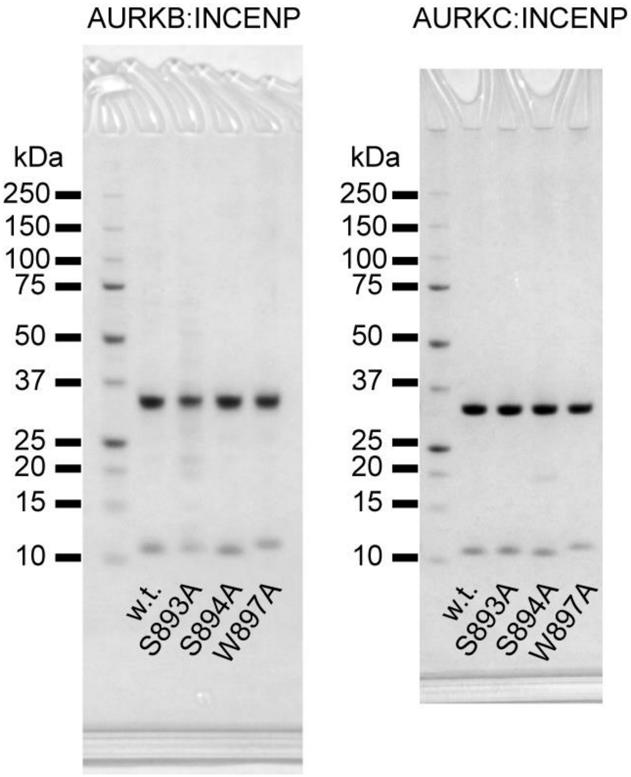

A 4-12% gradient gel showing the purified proteins used for enzymatic assays. Each lane contains the same total amount of protein as determined by absorbance at 280 nm. Expected MW: 33761.7 (AURKB), 31953.7 (AURKC), 8129.3 (INCENP w.t.), all excluding phosphorylation.

Supplementary References

1. Sievers, F., Wilm, A., Dineen, D., Gibson, T. J., Karplus, K., Li, W., Lopez, R., McWilliam, H., Remmert, M., Söding, J., Thompson, J. D., and Higgins, D. G. (2011) Fast, scalable generation of high-quality protein multiple sequence alignments using Clustal Omega. *Mol. Syst. Biol.* **7**, 539

2. Bond, C. S., and Schüttelkopf, A. W. (2009) *ALINE* : a WYSIWYG protein-sequence alignment editor for publication-quality alignments. *Acta Crystallogr. Sect. D Biol. Crystallogr.* **65**, 510–512

3. Bayliss, R., Sardon, T., Vernos, I., and Conti, E. (2003) Structural basis of Aurora-A activation by TPX2 at the mitotic spindle. *Mol. Cell*. **12**, 851–62

4. Sessa, F., Mapelli, M., Ciferri, C., Tarricone, C., Areces, L. B., Schneider, T. R., Stukenberg, P. T., and Musacchio, A. (2005) Mechanism of Aurora B activation by INCENP and inhibition by hesperadin. *Mol. Cell*. **18**, 379–91

5. Elkins, J. M., Santaguida, S., Musacchio, A., and Knapp, S. (2012) Crystal Structure of Human Aurora B in Complex with INCENP and VX-680. *J. Med. Chem.* **55**, 7841–7848
